# Spatiotemporal variation of the association between climate dynamics and HFRS outbreaks in Eastern China during 2005-2016 and its geographic determinants

**DOI:** 10.1371/journal.pntd.0006554

**Published:** 2018-06-06

**Authors:** Junyu He, George Christakos, Jiaping Wu, Bernard Cazelles, Quan Qian, Di Mu, Yong Wang, Wenwu Yin, Wenyi Zhang

**Affiliations:** 1 Ocean College, Zhejiang University, Zhoushan, China; 2 Department of Geography, San Diego State University, San Diego, California, United States of America; 3 Institute de Biologie de I’Ecole Normale Superieure UMR 8197, Eco-Evolutionary Mathematics, Ecole Normal Superieure, Paris, France; 4 International Center for Mathematical and Computational Modeling of Complex Systems (UMMISCO), UMI 209 IRD-UPMC, Bondy, France; 5 Center for Disease Surveillance of PLA, Institute of Disease Control and Prevention of PLA, Beijing, China; 6 Division of Infectious Diseases, Key Laboratory of Surveillance and Early-warning on Infectious Disease, Chinese Center for Disease Control and Prevention, Beijing, China; The University of Kansas, UNITED STATES

## Abstract

**Background:**

Hemorrhagic fever with renal syndrome (HFRS) is a rodent-associated zoonosis caused by hantavirus. The HFRS was initially detected in northeast China in 1931, and since 1955 it has been detected in many regions of the country. Global climate dynamics influences HFRS spread in a complex nonlinear way. The quantitative assessment of the spatiotemporal variation of the “HFRS infections-global climate dynamics” association at a large geographical scale and during a long time period is still lacking.

**Methods and findings:**

This work is the first study of a recently completed dataset of monthly HFRS cases in Eastern China during the period 2005–2016. A methodological synthesis that involves a time-frequency technique, a composite space-time model, hotspot analysis, and machine learning is implemented in the study of (a) the association between HFRS incidence spread and climate dynamics and (b) the geographic factors impacting this association over Eastern China during the period 2005–2016. The results showed that by assimilating core and city-specific knowledge bases the synthesis was able to depict quantitatively the space-time variation of periodic climate-HFRS associations at a large geographic scale and to assess numerically the strength of this association in the area and period of interest. It was found that the HFRS infections in Eastern China has a strong association with global climate dynamics, in particular, the 12, 18 and 36 mos periods were detected as the three main synchronous periods of climate dynamics and HFRS distribution. For the 36 mos period (which is the period with the strongest association), the space-time correlation pattern of the association strength indicated strong temporal but rather weak spatial dependencies. The generated space-time maps of association strength and association hotspots provided a clear picture of the geographic variation of the association strength that often-exhibited cluster characteristics (e.g., the south part of the study area displays a strong climate-HFRS association with non-point effects, whereas the middle-north part displays a weak climate-HFRS association). Another finding of this work is the upward climate-HFRS coherency trend for the past few years (2013–2015) indicating that the climate impacts on HFRS were becoming increasingly sensitive with time. Lastly, another finding of this work is that geographic factors affect the climate-HFRS association in an interrelated manner through local climate or by means of HFRS infections. In particular, location (latitude, distance to coastline and longitude), grassland and woodland are the geographic factors exerting the most noticeable effects on the climate-HFRS association (e.g., low latitude has a strong effect, whereas distance to coastline has a wave-like effect).

**Conclusions:**

The proposed synthetic quantitative approach revealed important aspects of the spatiotemporal variation of the climate-HFRS association in Eastern China during a long time period, and identified the geographic factors having a major impact on this association. Both findings could improve public health policy in an HFRS-torn country like China. Furthermore, the synthetic approach developed in this work can be used to map the space-time variation of different climate-disease associations in other parts of China and the World.

## Author summary

China has the largest number of HFRS infections in the world (9045 cases in 2016). Previous studies have found that HFRS infections are related to climate. However, the spatiotemporal distribution of the association between HFRS outbreaks at a large scale and global climate dynamics (i.e., over Eastern China during the period 2005–2016), as well as the identification of the geographic factors impacting this association have not been studied yet. This is then the dual focus of the present study. Strong synchronicities between global climate change and HFRS infections were detected across the entire study area that were linked to three main time periods (12, 18 and 36 mos). Specifically, strong and weak associations with non-point effects were detected in the south and middle-north parts of the study region, respectively. The climate impacts on HFRS were becoming increasingly sensitive with time. On the other hand, the geographic location (north coordinate, distance to coastline, east coordinate) makes a considerable contribution to the climate-HFRS association. As regards land-use, grassland and woodland were found to play important contributing roles to climate-HFRS association. Certain space-time links between global climate dynamics and HFRS infections were confirmed at a large spatial scale and within a long time period. The above findings could improve both the understanding of the HFRS transmission pattern and the forecasting of HFRS outbreaks.

## Introduction

Hantaviruses are RNA viruses that belong to the Hantaviridae family. Hantavirus infection causes hemorrhagic fever with renal syndrome (HFRS) to humans. As a rodent-borne infectious disease, the main domestic animals in China carrying hantavirus (including *Hantaan* virus, HTNV, and Seoul virus, SEOV) are *Apodemus agrarius* and *Rattus norvegicus* [1, 2]. Specifically, HTNV can survive for more than 96 days outside the host’s body under wet conditions at a temperature of 4°C [3]. Having a high viability in the environment, the viruses can be transmitted to humans by contacting to virus contaminated material, such as inhalation of aerosols generated by urine or saliva, ingestion of infected food, or directly by rodents bites [4]. It has been reported that the HFRS death rate in China was 2.89% during the years 1950–2014 [5]. In China, during the period 1998–2007 the number of male patients was three times higher than that of female patients, 87.32% of the documented HFRS cases were 15 to 60 years old, and 70% of them were farmers [6]. HFRS remains a major concern in China, because, although a declining HFRS trend has been observed at a global scale in China, there still exist certain local regions that continue to display increasing HFRS trends [7].

The HFRS infections exhibit a well-defined annual cycle that corresponds to the local variability of climate factors, anthropogenic activity and land-use change [8–10]. Specifically, a trophic cascade has been found between local climate and HFRS infections, i.e., the local precipitation and temperature affecting the living environment and primary food production, also contribute to the growth of the rodent population and the probability of interaction between infected rodents and humans [11]. To explore the climate effects on HFRS infections, previous studies have used quantitative techniques that explicitly incorporate climate factors in the estimation of the number of HFRS cases or the disease incidence. For example, Li et al. [12] employed a seasonal autoregressive integrated moving average model and found that the HFRS cases in Heilongjiang Province were closely associated with relative humidity, maximum temperature, and the southern oscillation index. Using a structure equation model, Guan et al. [13] observed that the HFRS incidence in Huludao City was correlated with temperature, air pressure, virus-carrying index, precipitation and relative humidity; using a Bayesian time-series Poisson adjusted model was found that the HFRS outbreak was related to preceding rainfall of 2–3 months ago [14]. Moreover, the principal components regression model, the multivariate polynomial distributed lag model, and the Poisson regression model have been used to explore climate-HFRS associations in Shenyang City, Chenzhou City, and the Elunchun and Molidawahaner counties, respectively [15–17]. Local characteristics of these associations were investigated in these studies, specifically the climate factors with considerable contributions to human HFRS infections (including the El Niño index). However, none of these studies was concerned with the large-scale investigation of the variation of the climate-HFRS association. Hence, a systematic quantitative study of the climate-HFRS associations at a large space-time scale is still lacking.

As a global climate phenomenon, El Niño-Southern Oscillation (ENSO) has been found to have a large impact on local precipitation and temperature [18, 19] and to affect local ecological conditions and animal lives, including disease-related rodents [20]. It has been suggested that ENSO played a significant role in driving the inter-annual variation of rodent- or vector-borne diseases, such as dengue fever and hantavirus cardiopulmonary syndrome [21–25]. Moreover, in previous studies ENSO has exhibited a multiannual variability, whereas a significant variation of multiannual periodicities has been reported for HFRS at several small regions of China [11, 26, 27]. Therefore, it is interesting to explore the internal association (co-variation) between ENSO and HFRS. Considering its global impact on climate, ENSO is regarded as a global climate dynamics index in the investigation of climate-HFRS associations. Although earlier studies showed that wavelet analysis is a powerful tool in handling non-stationary time series [28], it cannot individually handle simultaneously several time series distributed at a large spatial scale, which is the case of the Chinese HFRS data of interest in this work. On the other hand, Bayesian maximum entropy (BME, [29, 30]) is a powerful data modeling approach that can jointly assimilate any number of time series at various spatial locations by means of spatiotemporal random field modeling. BME integrates the available core (or general) knowledge about the effects of global climate dynamics on local HFRS infections with site-specific information in a realistic space-time domain. It is a versatile quantitative method that can study non-stationary, non-linear and non-Gaussian systems, which is why it has been successfully used in many scientific disciplines, such as environmental sciences, ecology, public health and epidemiology (for a review, see, e.g., [31]).

In view of the above considerations, the present work proposes a synthetic quantitative approach to study the spatiotemporal variation of the “global climate dynamics-HFRS” association in Eastern China under conditions of in-situ uncertainty. This approach has four main components: (a) Wavelet coherency analysis is used to assess quantitatively the association between global climate dynamics and HFRS at each city. (b) BME is used to estimate the strength of this association at a large domain and depict its spatiotemporal characteristics in terms of detailed maps. (c) The geographic boundaries of strong *vs*. weak associations are determined by hotspot analysis. (d) Lastly, a gradient boosting machine (GBM) model is used to investigate the specific impacts of the relevant geographic information on the global climate dynamics-HFRS association.

## Materials and methods

### Data collection

#### Ethics statement

The present study was approved by Institute of Disease Control and Prevention and Chinese Center for Disease Control and Prevention. All the HFRS data were anonymously analyzed for the consideration of confidentiality.

#### The HFRS dataset

**Fig 1:**
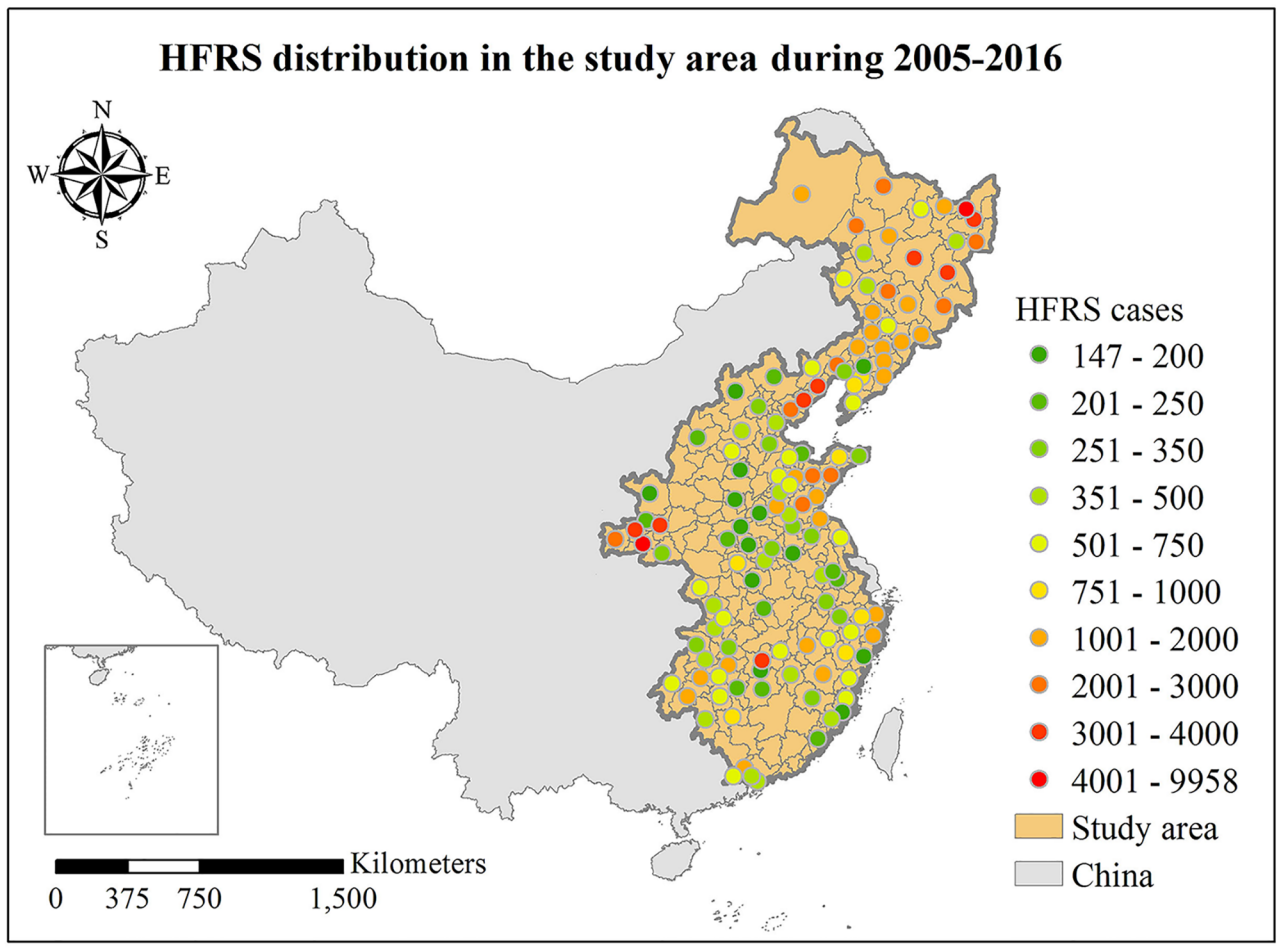
Distribution of HFRS cases in the study area during the period 2005–2016.

Yan et al. [32] have reported that approximately 86.4% of the total number of HFRS cases occurred in Eastern China and the Sichuan basin. Accordingly, in this study we used a recently completed dataset consisting of monthly HFRS cases at 127 cities in Eastern China during the period of January 2005-December 2016 collected by the China Information System for Disease Control and Prevention (CISDCP). These cities are distributed in 19 provinces, autonomous regions and metropolitan areas in Eastern China with a total area of approximately 2,820,000 Km^2^, including Beijing, Tianjin, Inner Mongolia, Heilongjiang, Jilin, Liaoning, Hebei, Henan, Shaanxi, Shanxi, Shandong, Hubei, Hunan, Anhui, Jiangsu, Zhejiang, Jiangxi, Fujian, and Guangdong, see Fig 1 (the corresponding HFRS incidence map covering 127 cities can be found in S1 Fig). Fig 1 shows that the northeastern and western parts of the study region have a high number of HFRS infection cases (the quantitative analysis in this work was performed in terms of the number of HFRS cases).

#### Multivariate El Niño southern oscillation index

The multivariate El Niño southern oscillation index (MEI) is calculated in terms of six oceanic and meteorological variables, namely, sea level pressure, zonal and meridional components of surface wind, sea surface temperature, surface air temperature, and total cloudiness fraction of sky. As regards MEI interpretation, large positive MEI values indicate the presence of El Niño phenomena, whereas large negative MEI values indicate the presence of La Nina phenomena [33]. Accordingly, MEI is regarded as a proxy describing global climate dynamics quantitatively, especially in terms of worldwide temperature and precipitation levels [34]. Since climate has been associated with HFRS spread and outbreaks, in this work we used the MEI to explore the association between HFRS distribution in China and the global climate dynamics. The MEI dataset is available at http://www.esrl.noaa.gov/psd/enso/mei/table.html.

#### Geographic data

During 2015, in the region of interest six land-use types were considered: cropland, woodland, grassland, water, urban, and barren (see S2 Fig). At each location of interest, the number of pixels for each land-use type within a 50 Km radius buffer was calculated using the ArcGIS 10.2 software (each pixel represents an area of 1 Km^2^). Distances to coastline and elevations were obtained by means of the national administrative map and a digital elevation model, respectively. All the geographic data were provided by the Data Center for Resources and Environmental Sciences of the Chinese Academy of Sciences (RESDC; http://www.resdc.cn).

### Synthetic methodological framework

S3 Fig presents an outline of the synthetic methodological framework used in this work. More detailed information about the various components of this framework is given below.

#### Wavelet coherency analysis

The association between global climate dynamics and HFRS cases at each Chinese city was measured by the mathematical “magnifying glass” of wavelet coherency analysis [35, 36]. This method expresses quantitatively the internal co-variation between two time series in terms of the synchronicity strength of the trends of the two series. In the present study this implies that the larger the coherency value is, the stronger is the association between global climate dynamics and HFRS infections. The month-to-month time series pairs (HFRS cases at a city and corresponding MEI) were used to implement wavelet coherency analysis in three steps: (1) the two time series were separately transformed by Morlet wavelets, (2) the two wavelet-transformed series were subsequently cross-wavelet transformed to obtain the wavelet cross-spectrum, and (3) the wavelet coherency was finally derived by using the spectrum of each series to normalize the cross-spectrum as follows,

$$W(\tau ,\alpha )=\frac{1}{\sqrt{\alpha }}\int_{-\infty }^{+\infty }Z(t)\;\psi^{*}(\frac{t-\tau }{\alpha })dt,$$ (1a)

$$W_{Z_{1}Z_{2}}=W_{Z_{1}}W_{Z_{2}}^{*},$$ (1b)

$$C_{Z_{1}Z_{2}}(\tau ,\alpha )=\frac{\left\|\langle W_{Z_{1}Z_{2}}(\tau ,\alpha )\rangle \right\|}{{\left\|\langle W_{Z_{1}Z_{1}}(\tau ,\alpha )\rangle \right\|}^{1/2}{\left\|\langle W_{Z_{2}Z_{2}}(\tau ,\alpha )\rangle \right\|}^{1/2}},$$ (1c)

where *Z*_1_ and *Z*_2_ denote the MEI and HFRS case series, respectively, *Ψ*(·) is the Morlet wavelet, *α* and *τ* represent the scale factor and time shift, respectively, and the *, <·> and ||·|| denote the complex conjugate, smoothing and modulus operator, respectively. The larger the function $C_{z_{1}z_{2}}$ in Eq (1c) is, the stronger is the coherency of the time series *Z*_1_ and *Z*_2_, i.e., the HFRS infections are more closely linked to global climate dynamics. The three steps above were repeated 127 times (one for each city) to obtain the coherency between MEI and HFRS cases at each city. The mean wavelet coherency spectrum was calculated at each province (autonomous region and metropolitan area) to better understand the varying impacts of climate dynamics on HFRS infections in various parts of Eastern China.

#### Spatiotemporal mapping of the coherence strength between MEI and HFRS cases

At each city a separate coherency time series can be generated to characterize the temporal variability of the MEI-HFRS association strength. The association between MEI and HFRS was visualized quantitatively with the help of the specific bands (high values) of the wavelet coherency spectra at the various cities. The mean and variance of wavelet coherency at all cities were derived from the spectra across the time-frequency domain. Subsequently, two criteria were used for selecting the global character bands: high mean wavelet coherency values in the character bands represent a strong MEI-HFRS association, and not very small variances within the same bands capture the variability of this association at various cities. Given the selected bands, the mean value of the corresponding band coherency values at each time instance can be obtained at each city to construct space-time coherency data set.

The BME theory was used to estimate the coherency (strength of MEI-HFRS association), *C*(***p***), as a function of the point ***p*** = (***s***,*t*) in the space-time domain of interest (the vector ***s*** denotes spatial coordinates and the scalar *t* denotes time). In BME theory, a spatiotemporal random field model (S/TRF; [37, 38]) represents the composite geographic-chronological variation of the coherency values in conditions of in situ uncertainty. The fact that coherency *C*(***p***) is modeled as an S/TRF implies that it is mathematically described by a probability density function (pdf) *f*_*C*_(***p***; *χ*), where *χ* is a possible S/TRF realization (coherency value) at space-time point ***p*** with probability of occurrence determined by the pdf. In this case, BME constructs the pdf by integrating the core (or general) knowledge base (*G*-KB, consisting of the theoretical *C*(***p***) mean and covariance models) and the site-specific knowledge base (*S*-KB, including the mean coherency values). Note that only hard data (i.e., coherency values) were considered as the site-specific knowledge base in this study. The basic set of BME equations of spatiotemporal coherency modeling and mapping are

$$\int d\chi \;(g-\bar{g})\;e^{\mu \cdot g}=0,$$ (2a)

$$\int d\chi \;\xi \;e^{\mu \cdot g}=a\;f_{C},$$ (2b)

where *α* is a normalization parameter, ***g*** represents the available *G*-KB, $\bar{g}$ denotes the expected value of ***g***, ***μ*** are coefficients expressing the relative importance of ***g*** so that ***μ*** · ***g*** = Σ_*i*_*μ*_*i*_*g*_*i*_, and *ξ* represents the *S*-KB (technical details can be found in the relevant literature; e.g., [31]; and references therein). By solving the above set of equations, the coherency values at unsampled points on a 10*km* × 10*km* × 1*mo* space-time grid covering the study area and period of interest were estimated by BME for mapping purposes. In particular, the SEKS-GUI software library [39] was selected to produce these space-time coherency maps. Using a 10-fold cross validation technique, the BME performance was subsequently evaluated in terms of three accuracy indicators: the mean absolute error (MAE), the root mean squared error (RMSE), and the *R*^2^ of the simple linear regression model relating observed and predicted values.

#### Hotspot analysis of the coherency strength maps

Due to the fact that climate exerts non-point impacts on HFRS infections, the coherency values between HFRS and climate dynamics are expected to exhibit local cluster characteristics. In this case, hotspot analysis can efficiently assess high- *vs*. low-valued coherency clusters across space [40], i.e., it can identify strong *vs*. weak associations between global climate dynamics and HFRS infected areas. Specifically, a hot spot means that the coherency values at a given and the neighboring locations are all high; naturally, a low spot means the opposite, i.e., the coherency values at a given and the neighboring locations are all low. In Arcgis 10.2, the functions (Getis-Ord Gi*) of hotspot analysis are as follows,

$$G_{i}^{*}=\frac{\sum_{j=1}^{n}w_{i,j}c_{j}-\bar{C}\sum_{j=1}^{n}w_{i,j}}{S\sqrt{\frac{n\sum_{j=1}^{n}w_{i,j}^{2}-{\left(\sum_{j=1}^{n}w_{i,j}\right)}^{2}}{n-1}}},$$ (3a)

$$\bar{C}=\frac{\sum_{j=1}^{n}c_{j}}{n},$$ (3b)

$$S=\sqrt{\frac{\sum_{j=1}^{n}c_{j}^{2}}{n}-{\bar{C}}^{2}},$$ (3c)

where *c*_*j*_ is the coherency value at location *j*, $\bar{C}$ and *S* denote, respectively, the mean and variance of the coherency values at all locations, *n* is the total number of locations, and *w*_*i*,*j*_ is the spatial weight between locations *i* and *j*. By using the corresponding tool “Hot Spot Analysis (Getis-Ord Gi*)” in ArcGIS 10.2, hot *vs*. cold spots were determined and mapped at 0, 90, 95, and 99% significance level. In general, the spatial heterogeneity of a map can be either local or stratified. Hotspot analysis provides information about local spatial heterogeneity. In this study, we also employed *q*-statistics to test the spatial stratified heterogeneity [41].

#### Gradient boosting machine

The gradient boosting machine (GBM; [42, 43]) is a very flexible machine learning approach that is highly customizable to the particular needs of the study of interest [44]. One of the attractive GBM features, compared to other statistical classifiers, is that the relative importance of an explanatory variable can be determined and the partial dependence of each variable can be obtained [42]. After a number of iterations, the GBM selects an optimal function $y=\hat{F}\left(x\right)$ relating the explanatory variables denoted by the vector ***x*** with the response variable *y* by minimizing the loss function *L*(*y*,*F*(***x***)). Then, the basic GBM equations are

$$F_{t}(x)=F_{t-1}(x)+\beta_{t}h(x;a_{t}),$$ (4a)

$$a_{t}=\text{arg}\underset{a,\rho }{\text{min}}\sum_{i=1}^{N}\left[-\frac{\partial L[y_{i},F(x_{i})]}{\partial F(x_{i})}{|}_{F(x)=F_{t-1}(x)}-\rho h(x_{i};a)\right],$$ (4b)

$$\beta_{t}=\text{arg}\underset{\beta }{\text{min}}\sum_{i=1}^{N}L[y_{i},F_{t-1}(x_{i})+\beta h(x_{i};a_{t})],$$ (4c)

where *t* = 1,2,…,*K* denotes the interaction time, *N* is the number of data, *h*(***x***;***a***) is a function called the “base learner” with vector parameter ***a*** = (*a*_1_,*a*_2_,…) that can be calculated by solving Eq (4b). Technically, *h*(***x***;***a***) is usually considered as an *L* terminal node regression tree, *β* are expansion coefficients calculated by solving Eq (4c) so that after *K* iterations the final function *F*(***x***) is obtained. Specifically, cropland, woodland, grassland, water, urban, barren (in a 50km buffer), elevation, distance to coastline, and spatial coordinates (using Krasovsky 1940 Albers projection in ArcGIS 10.2) were selected as the explanatory variables for assessing the coherency maps obtained by BME. This process was computationally implemented by using the "gmb" package of the R statistics software. Similarly, the 10-fold cross validation technique was employed to test the performance of the GBM model in terms of MAE, RMSE and *R*^2^.

## Results

### Wavelet coherency analysis

**Fig 2:**
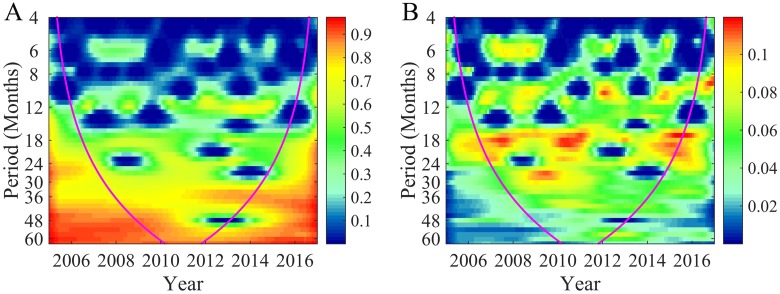
Global statistics of wavelet coherency spectra at all Chinese cities considered. **A** Mean wavelet coherency spectrum, and **B** variance of wavelet coherency spectrum at all cities. Purple line represents the influence cone that delimits the region that is totally not influenced by edge effects.

In total, 127 wavelet coherency spectra were obtained (one at each city in Eastern China) by means of wavelet coherency analysis (S4 Fig). By averaging the wavelet coherency spectra in each Chinese province and autonomous region, the mean wavelet coherency spectra at 19 provinces, autonomous regions and metropolitan areas were calculated and plotted in S5 Fig. These figures indicate that the closer the provinces are, the more similar are the corresponding wavelet coherency figures. For illustration, S5B–S5D Fig show that the shapes of the wavelet coherency plots are almost the same, and the only small differences visually observed are in the coherency values. At the three provinces (Heilongjiang, Jilin, Liaoning) strong coherencies (high coherency values shown in S5B–S5D Fig) exist between HFRS and global climate dynamics during three main period bands, i.e., 12, 18 and 36 mos. Among the three provinces, Heilongjiang has the largest coherency value during the 36 mos band, as displayed in S5B Fig. Wavelet coherency analysis thus confirmed quantitatively that the association between HFRS cases and global climate dynamics shows a strong coherency during the 3-yrs (36-mos) multiannual oscillations of the entire period 2005–2016; this phenomenon is also observed in terms of the global statistics of Fig 2. The same three period bands (12, 18 and 36 mos) with high coherency values can be detected in terms of the characteristic bands of Fig 2A, which depict the inter-association between HFRS cases and global climate dynamics. Furthermore, by comparing the coherency variances at all cities in Fig 2B, we found that the coherency during the 36-mos band has the lowest variance among the three main bands, thus suggesting that the HFRS-climate association is more consistent during the 36-mos band. Therefore, the 30–42 mos bands were selected as coherency character bands expressing HFRS-climate associations for further analysis.

### Spatiotemporal mapping of the association between HFRS and global climate dynamics

Given the selected character bands (30–42 mos), the mean coherency values for the entire 2005–2016 period were calculated at each city (thus providing the space-time coherency dataset needed for further processing). This dataset was then analyzed using the SEKSGUI software library [39]. The calculated empirical covariance values shown in S6 Fig imply that the coherency between HFRS and global climate dynamics is spatially dependent and temporal sustained. To the empirical covariance values we fitted the theoretical space-time model of coherency variation

$$c_{C}(|h|,\tau )=0.88[1-\frac{3|h|}{2\times 10^{5}}+\frac{1}{2}{\left(\frac{\left|h\right|}{10^{5}}\right)}^{3}]\text{exp}[-{\left(\frac{\sqrt{3}\tau }{40}\right)}^{2}]+0.12[1-\frac{3|h|}{4.4\times 10^{6}}+\frac{1}{2}{\left(\frac{\left|h\right|}{2.2\times 10^{6}}\right)}^{3}]\text{exp}[-{\left(\frac{\sqrt{3}\tau }{72}\right)}^{2}],$$ (5)

where |***h***| and *τ* denote the spatial distance (in meters) and temporal separation (in months), respectively. The numerical 10-fold cross validation results confirmed that BME generates accurate coherency estimates (the corresponding accuracy indicators were *R*^2^ = 0.991, *MAE* = 0.00271, and *RMSE* = 0.0139). Hence, the SI section presents several of these BME-generated spatiotemporal maps that offer a detailed picture of the strength of the climate-HFRS association in Eastern China for each month of the period 2005–2016 (144 maps, in total).

**Fig 3:**
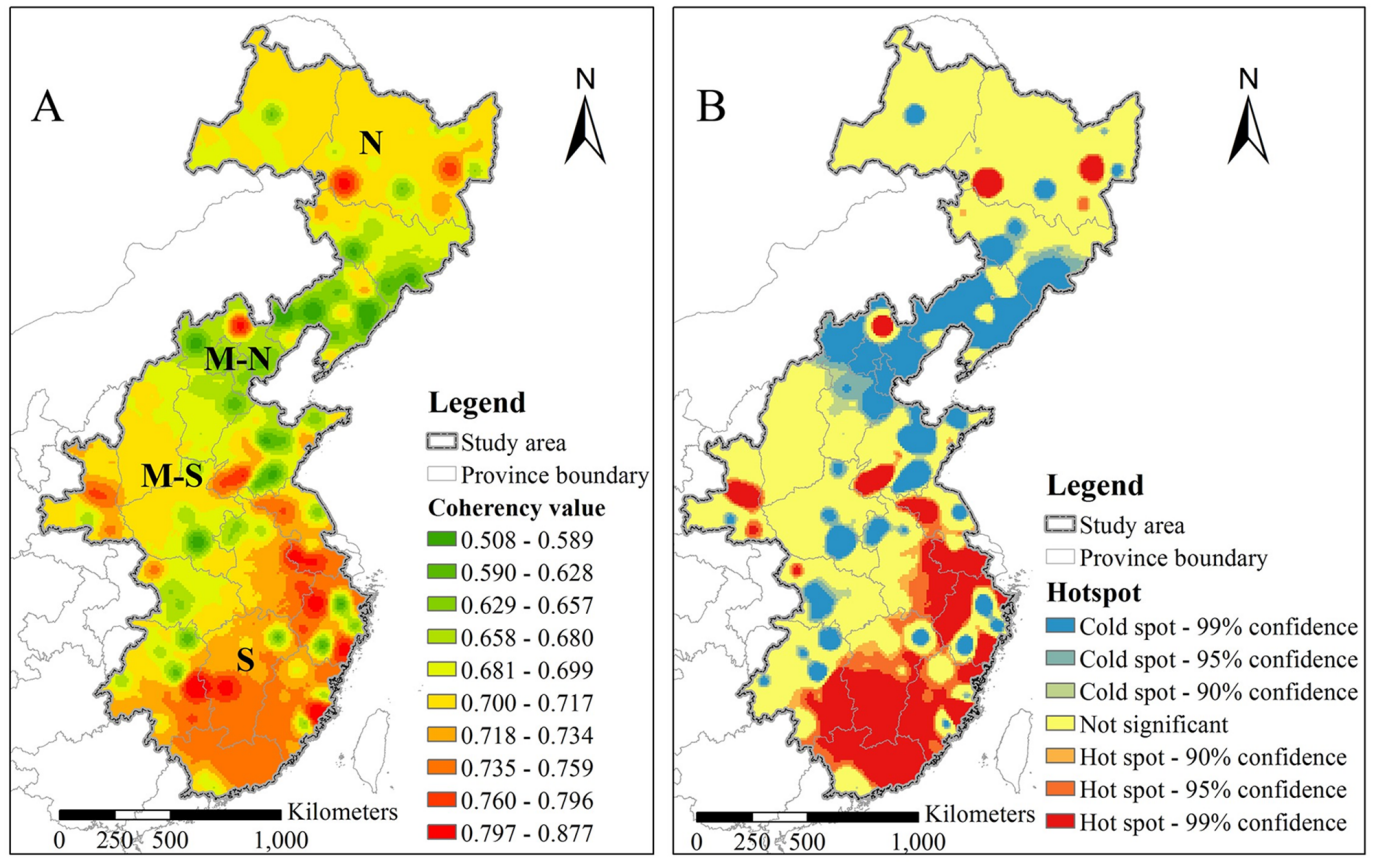
Association between HFRS cases and global climate dynamics. **A** Map of the association strength, and **B** map of the association hot spots. The N, M-N, M-S and S represent the north, middle-north, middle-south and south parts of the study region, respectively.

To avoid the edge-effects of wavelet coherency analysis at the first and last year, the 120 maps of the period 2006–2015 were used to explore further the climate-HFRS association pattern across the study area (Fig 3A). Based on the calculated coherency values, the region can be divided vertically into four parts (south, middle-south, middle-north and north parts) suggesting that local characteristics can affect the association. In particular, the south and middle-north parts have the highest and the lowest coherency values, respectively, whereas the other two parts exhibit mediocre coherency values. A few low coherency sections were observed in the south, middle-south and north parts of the study region. In S8–S17 Figs, several cities were closely associated with global climate dynamics during the entire period 2006–2015 with consistently high coherency values. Moreover, the presence of an upward climate-HFRS coherency trend during the period 2013–2015 indicated that the level of climate impacts on the HFRS disease is becoming increasingly sensitive during this period (see S15–S17 Figs). Notice that the coherency values at several locations increased during 2014 implying that the HFRS infections at these locations became more closely associated with global climate dynamics than during previous years (S16 Fig).

For the entire study region, the spatial heterogeneity of Fig 3A is weak, implying that both high values and low values are clustered geographically. This phenomenon is tested by hotspot analysis. In fact, more obvious clusters can be found in Fig 3B, see the geographic distribution of the hot and cold spots corresponding to the HFRS-global climate association map of Fig 3A (the distribution of the spots in Fig 3B delimits geographically high- and low-valued clusters). Actually, compared to Fig 3A and 3B makes it much easier to define visually the boundaries of the hot and cold spots. Four distinct parts of the HFRS-global climate association pattern can be detected in these figures, including the south, middle-south, middle-north and north parts of the study area. Most clusters with high association coherency (i.e., hot spots) are located in the south part of the study area, whereas most clusters with low coherency (cold spots) are located in the middle-north part. Notice that some hot spots are close to each other, and the same is true for some cold spots. As a result, a large spatial continuous hop spot area as well as a large cold spot area are generated; e.g., see the south and the middle-north parts of Fig 3B. The other two parts don’t exhibit any noticeable distributions of continuous non-point hot/cold spots. Yet, a number of point hot/cold spots can be still found at certain places of the study area. The corresponding monthly hotspot maps are displayed in the SI section of this work (S31 and S32 Figs).

Furthermore, the spatial stratified heterogeneity revealed in Fig 3 was tested in terms the *q*-statistic. The result showed that the maps of Fig 3 exhibit strong stratified heterogeneity (*q* = 0.80 with significance 1.68E-09). The same process was applied in S7–S30 Figs, in which cases it was found that the *q*-values ranged from 0.65 to 0.84 with the associated significance ranging from 1.18E-09 to 2.00E-09 (S33 Fig).

### Impact of geographic factors on the association between HFRS and global climate dynamics

Multiple linear regression modeling was initially used to test the relationship between the ten explanatory variables introduced above (i.e., cropland, woodland, grassland, water, urban, barren, elevation, distance to coastline and spatial coordinates) and the response variable (association strength). The results showed that all ten variables have a significant influence on the climate-HFRS association (in all ten cases, it was found that *p* < 0.05; see S1 Table), whereas the *R*^2^ value of linear regression was equal to 0.30 with *p* < 2.2 × 10^−16^.

**Fig 4:**
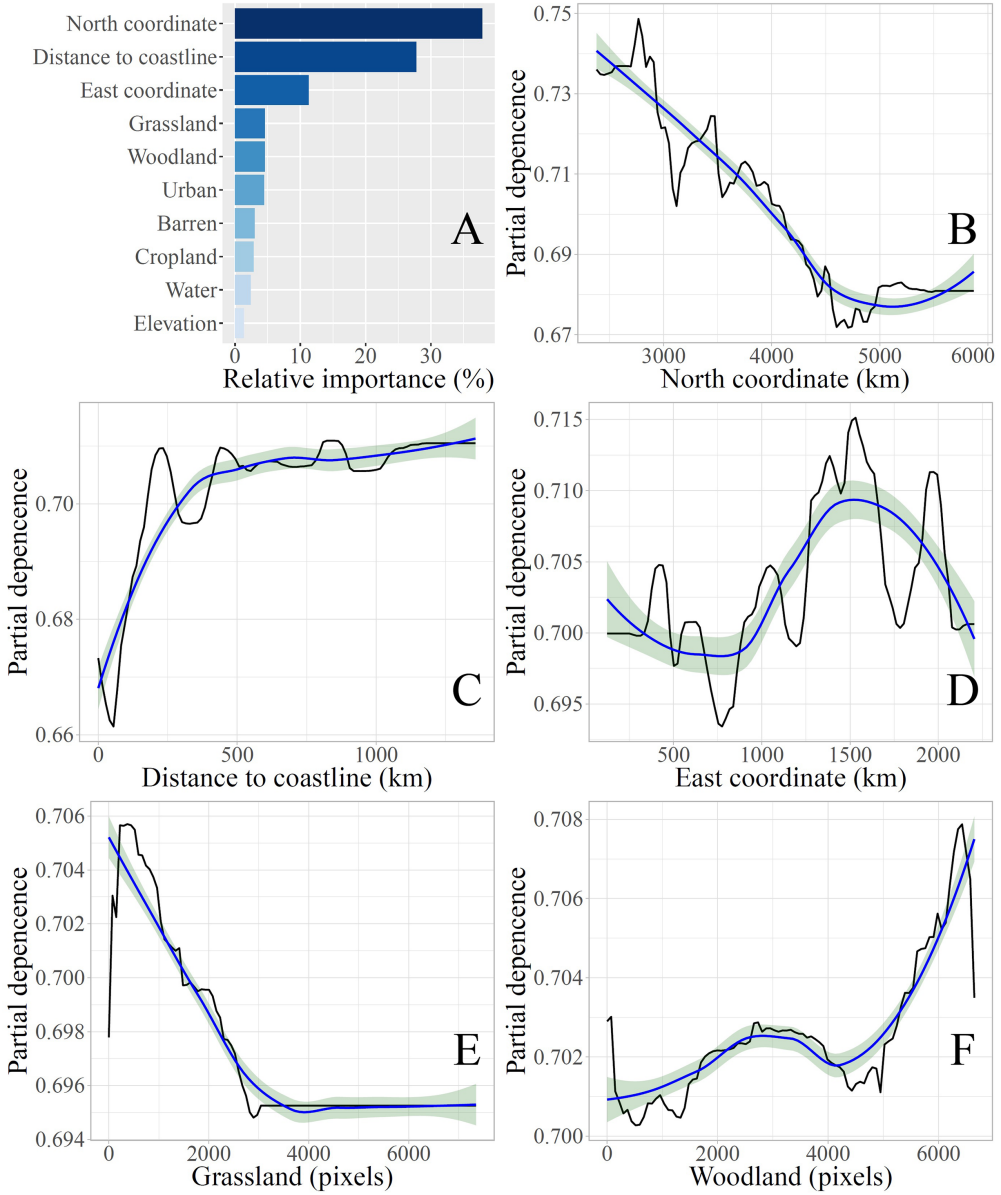
Relative importance of various explanatory variables (**A**) and the partial dependence (black lines) of the five most important variables (i.e., north coordinate, distance to coastline, east coordinate, grassland, woodland, **B-F**) of the GBM model. Blue lines denote partial dependence trends calculated by local polynomial regression with the surrounding shadow indicating the 95% confidence interval.

All explanatory variables together with the response variable were used to construct the GBM model. The performance of the model was evaluated in terms of a 10-fold cross validation method. It was found that the accuracy indicator values were *R*^2^ = 0.94, *MAE* = 8.71 × 10^−5^ and *RMSE* = 9.33 × 10^−3^, which is a much better performance than that of the multiple linear regression model. Fig 4A shows the relative importance of the ten explanatory variables on the climate-HFRS association obtained by the GBM model. The five most important explanatory variables were the “north coordinate” (latitude), “distance to coastline”, “east coordinate” (longitude), “grassland” and “woodland” with relative importance scores 37.84%, 27.75%, 11.29%, 4.56%, 4.55%, respectively. The corresponding partial dependence plots for the “north coordinate”, “distance to coastline”, “east coordinate”, “grassland” and “woodland” are displayed in Fig 4B–4F. These partial dependence plots show the effect of each geographic factor on the climate-HFRS association after accounting for the average effects of all other factors in the GMB model. An apparent negative relationship was detected between the “north coordinate” and the climate-HFRS association (Fig 4B), i.e., the more northward is located the city the weaker is the climate-HFRS association (this is especially valid in the south part of the study region depicted in Fig 3A). On the other hand, an increasing trend with local fluctuations was found between “distance to coastline” and the climate-HFRS association (Fig 4C). The nonlinear (wave-like) variation effect of the “east coordinate” on the climate-HFRS association was clearly revealed in the plot of Fig 4D (e.g., the smooth trend of the “east coordinate” has a global sine shape with local wave fluctuations). The partial dependence of grassland exhibits a rapid increase-decrease-stable trend as a function of the grassland area (Fig 4E). The positive relationship between the climate-HFRS association and woodland is shown in Fig 4F. The other partial dependence plots can be found in the SI section (S34 Fig). Furthermore, we also constructed another GBM^#^ model in the SI section (the symbol “#” was used to distinguish it from the GBM model discussed above) that excluded the spatial coordinates of the ten explanatory variables in order to avoid possible interactions of the geographic factors with spatial coordinates and to obtain additional insight about the geographic factors effects on the climate-HFRS association. Comparing the results in SI (S1 Text and S35 Fig) with the plots of Fig 4 above, similar conclusions can be drawn about the effects of the geographic factors investigated by the GBM^#^ and by the GBM models.

## Discussion

Public health scientists and officers are concerned with questions like: “Are the HFRS outbreaks in a geographical region associated with global climate dynamics?” “How global climate dynamics affects the HFRS transmission pattern at a large spatial scale?” “Which geographic factors have significant impacts on the climate-HFRS association?” Answers to these and similar questions can offer valuable information for HFRS early warning, monitoring, and control purposes. The present work developed a novel synthetic quantitative analysis that can help answer these scientific questions. Methodologically, this approach is based on an integration of wavelet analysis, Bayesian maximum entropy, hotspot analysis and gradient boosting machine techniques. To the best of our knowledge, this is the first study that uses such a synthetic framework to assess climate-HFRS associations at a large geographic scale (Eastern China, covering an area of about 2.8 million Km^2^) and during a relatively long time period (2005–2016). The study is characterized by the originality of the HFRS dataset and the large amount of local and regional information available about several features of the climate-HFRS association in the time-frequency and the space-time domains.

A main outcome of the present study is the successful quantitative investigation of the association between global climate dynamics and human HFRS infections by wavelet analysis. Wavelet analysis uses the global climate index MEI (a proxy expressing global climate dynamics quantitatively) that has been found to be suitable for large-scale analysis of ecological processes [45]. It has been postulated in the literature that climate affects HFRS infections by influencing rodent-borne physiology and interaction in a complex system [46, 47]. For example, high positive MEI values infer the presence of El Niño phenomena, leading to higher precipitation levels in southeastern China during the months of December through May [48]. Hence, sufficient precipitation will help the reproduction of rodents, directly impacting the probability of rodent-human contacts and human infections. In this work, it was found that the large-scale HFRS surveillance dataset collected at 127 cities in Eastern China during 2005–2016 and the global climate dynamics records available exhibit a strong synchronicity in multiannual cycles, including 1, 1.5 and 3 yrs periods (Fig 2, S4 and S5 Figs). This finding provides strong quantitative support to an earlier claim that HFRS infections are closely associated with global climate dynamics through complicated nonlinear dynamics, including multiannual and seasonal variational patterns of both climate dynamics and rodent population [20, 49–51].

Another outcome of this work is related to the fact that wavelet analysis detected a strong association coherence between global climate dynamics and HFRS infections in Eastern China at 3 yrs cycle, indicating that both climate index and HFRS infections possess character multi-cycles with a 3 yrs period [36]. In this sense, this work confirmed at a large scale (Eastern China), what previous studies have observed at a local scale, particularly, in Changsha city, Xi’an city and Pingyi county the HFRS variation was found to be characterized by 1 and 3 yrs cycles [14, 26, 52]. Public health officers may appreciate this work’s finding that the corresponding wavelet coherency spectra in S4 Fig provide local information about the climate-HFRS association that can improve the understanding of the HFRS transmission pattern. Additionally, these results can be regarded as a general knowledge base for HFRS monitoring, controlling and forecasting purposes or for further research (e.g., global climate dynamics can serve as a potential predictor of the trends of human infections by HFRS).

We further explored the features of the climate-HFRS association by integrating information about the temporal variation of the association at Eastern China cities using the BME theory. The space-time variability of the climate-HFRS association represented quantitatively by the coherency covariance model showed a strong temporal dependence (S6A and S6C Fig), indicating that the climate-HFRS association exhibits low temporal variation, i.e., it remains stable locally. Moreover, a strong short-range spatial dependence with weak long-range heavy tails were also observed in the covariance model plots (S6A and S6B Fig). The interpretational implication of these covariance features is that the climate-HFRS association has different local characteristics than global synchronicity. The BME-generated maps of the spatiotemporal variation of the climate-HFRS association strength (coherency values) included high-resolution monthly maps (10Km × 10Km) covering the entire study area during the period 2005–2016 (S7–S18 Figs). In addition to the global findings of this work mentioned earlier, the composite spatiotemporal covariance plot of climate-HFRS association (S6 Fig) indicates the presence of a weak local heterogeneity combined with a strong stratified heterogeneity. In other words, clusters of high and low coherency values in the maps of climate-HFRS association reveal some interesting local features of the spatial variability of the climate-HFRS association.

At this point, another potentially significant finding was the presence of an upward climate-HFRS coherency temporal trend, especially during the period 2013–2015, which indicated that the climate impacts on HFRS in Eastern China were becoming increasingly sensitive with time. This phenomenon may be due to the fact that the frequency of extreme precipitation events shows a temporally increasing trend in the monsoon region of Asia [53, 54], which includes a large part of the study region. As has been reported in the relevant literature [55], winter temperatures are warming faster than summer temperatures, with the warm-event indices increasingly significantly with time (the temperature and precipitation effects on HFRS infections in the Eastern China region are further discussed below).

Public health officers may find it useful to study and evaluate, as appropriate, the geographical distribution of the climate-HFRS association in the BME-generated maps, like that of Fig 3A. The same is true as regards the strong and weak associations throughout Eastern China that are clearly outlined in the hot *vs*. cold spot map of Fig 3B. Hence, using such maps public health officers can assess, in quantitative terms, the strength of the climate-HFRS association at a specific location compared to the strength of the association at other locations (e.g., more attention should be paid to climate dynamics at hot spot locations). In a similar context, the health officers of a city in China may benefit by any effective HFRS control measures previously implemented in other cities with similar climate-HFRS association patterns.

In light of the above findings and inferences, an important further objective of this work was to investigate potential geographic determinants of the variation of climate-HFRS associations in Eastern China during 2005–2016. Generally, global climate dynamics is a macroscopic natural process at the earth scale exerting certain impacts on microscopic climate in local or regional domains with specific geographic features. In other words, local geographic information may either strengthen or weaken the link between global climate dynamics and HFRS infections by revealing local climatic conditions that can affect the rodents’ living environment [10, 11]. Accordingly, a machine learning technique, GBM, was employed to investigate the complex non-linear relationship between geographic factors and the climate-HFRS association in the Eastern China region. Interestingly, the most important determinants of the geographic variation of climate-HFRS association were found to be the spatial coordinates of a location and the distance to coastline (Fig 4A). If, e.g., an extreme (strong or weak) climate-HFRS association is detected at a specific location of Eastern China, it suggests that large-scale climate-driven effects dominate the association at this location. Notice that the geographical dependence of the climate-disease link has been observed in other parts of the World. Klempa [56], e.g., showed that in different parts of Europe climate dynamics affects hantavirus and its reservoir hosts in more than one ways.

Such findings would be valuable for local health management purposes, since, as was suggested earlier, health officers in hot spot areas should pay more attention to the global climate effects on HFRS transmission (this kind of information is known to help disease control and monitoring efforts, [57]). Notice that a location with a low “north coordinate” (latitude) is linked to a much stronger climate-HFRS association than one with a high “north coordinate”; Fig 4C and 4D reveal a simultaneous wave-like effect on the climate-HFRS association of the “distance to coastline” and the “east coordinate” (longitude), respectively. This observation suggests that HFRS outbreaks in coastal cities or riverbanks were particularly vulnerable to global climate dynamics (thus confirming in the Eastern China case a similar result obtained by Rosenzweig et al. [58]). Interestingly, as is shown in the SI section (S1 Text and S35 Fig), if the GBM^#^ model is used (that excludes spatial coordinates from the list of geographic factors under consideration) the partial dependencies of the geographic factors exhibit a few minor differences compared to the GBM model above. These findings deduced that interaction effects exist between the geographic factors, and that the GBM^#^ model can provide additional information concerning the possible effects of the geographic factors.

Yet another objective of this work was to study the way geographic factors impact climate-HFRS associations in Eastern China. We considered that a better investigation of the phenomenon is possible if the impacts of the geographic factors on the climate-HFRS association were divided into two parts, including impacts on climate and impacts on HFRS infections (rodent population). When the geographic factors have significant effects on both local climate and HFRS infections, they were expected to also impact the climate-HFRS association. Global climate dynamics drives local climate with various consequences (changing microclimatic conditions, including temperature, precipitation and evapotranspiration; see, [59]). For example, temperature variation over the western pacific region (including China) is controlled by ENSO [60]. In this work (Fig 4B), it was observed that global climate dynamics has more significant effects along the coastal regions of south China than in other parts of the country. In addition, global climate fluctuations can cause local precipitation variation [61]. Precipitation events will directly increase the primary food production for rodents, improve virus survival and stimulate rodent reproduction [62–64]. Precipitation can affect rodent population indirectly, by positively influencing the growth of grassland and woodland. Studies have shown that the normalized difference vegetation index NDVI can represent grassland and woodland, to a certain extent [65, 66]. It has also been proven that NDVI and the enhanced vegetation index (EVI) are highly correlated with rodent (deer mouse) density [67]. Hence, grassland and woodland have a significant contribution on the number of HFRS hosts and can further impact the climate-HFRS association (Fig 4E and 4F). Moreover, woodland can develop a stable ecosystem with strong stability and resilience during a low precipitation season [68, 69]. Specifically, woodland can improve water resource conservation or primary food production for rodents’ consumption, which also benefit the rodent population in a positive way and can further impact the climate-HFRS association. This is why the partial dependence of woodland shows a monotonically increasing trend as a function of woodland coverage (see Fig 4F). Beyond precipitation, warm winter temperatures can improve the survival of rodents [11], thus further impacting the climate-HFRS association. In this work, it was found that because of the higher winter temperatures occurring in the south part (with low “north coordinate”) than in the north part of the study area (thus, improving the rodents’ living conditions in the south compared to the north), there exists a stronger climate-HFRS association in the south with large-scale effects (Figs 3 and 4B). As a result, a growing rodent population will increase the probability of virus transmission among rodents, and, subsequently, will increase the probabilities of rodent-human contacts and HFRS infections among humans [14, 70]. In sum, the geographical factors have two main ways of affecting the climate-HFRS association: by impacting local climate and by directly impacting HFRS infections (these two ways are related, since they both enable the growth of local rodent populations which, in turn, increase the probability of rodent-human contact and infection). Hence, by taking these geographic factors into consideration, public health officers may improve their understanding of the climate-HFRS association.

It is hoped that the synthetic quantitative approach (developed in this work to map the space-time variation of climate-HFRS association in Eastern China) could be also applied in the study of different climate-disease associations in other parts of China or the world. Lastly, future work should be directed toward integrating the general knowledge and the site-specific information of the present study to forecast HFRS outbreaks at a large spatial scale covering the entire China.

## Supporting information

S1 Table Performance of the multiple linear regression model. (DOCX)

S1 Text The GBM^#^ model (excluding spatial coordinates). (DOCX)

S1 Fig Distribution of HFRS incidence in the study area during the period 2005–2016. Population in 2015 was used to standardize the HFRS cases in each city. The unit of HFRS incidence is cases/100,000 population.
(TIF)

S2 Fig Land use types of study area in 2015. (TIF)

S3 Fig Workflow of the synthetic methodological framework. (TIF)

S4 Fig Wavelet coherency spectra between global climate dynamics and HFRS infections at 127 cities in the study area. Purple line represents the cone of influence that delimits the region that is not influenced by edge effects; black line shows a = 5% significance level computed based on 500 bootstrap.
(GIF)

S5 Fig Zonal wavelet coherency spectra at 19 provinces, autonomous regions and metropolitan areas in the study area. **A–S** represent Inner Mongolia, Heilongjiang, Jilin, Liaoning, Beijing, Tianjin, Hebei, Shanxi, Shandong, Shaanxi, Henan, Jiangsu, Anhui, Hubei, Zhejiang, Hunan, Jiangxi, Fujian and Guangdong, respectively.
(TIF)

S6 Fig Empirical and fitted theoretical covariance model of climate-HFRS association. **A** Composite space-time empirical and fitted theoretical covariance; **B** empirical and fitted theoretical covariance when T-lag equals to 0; **C** empirical and fitted theoretical covariance when S-lag equals to 0.
(TIF)

S7 Fig Strength of the climate-HFRS association in 2005. (TIF)

S8 Fig Strength of the climate-HFRS association in 2006. (TIF)

S9 Fig Strength of the climate-HFRS association in 2007. (TIF)

S10 Fig Strength of the climate-HFRS association in 2008. (TIF)

S11 Fig Strength of the climate-HFRS association in 2009. (TIF)

S12 Fig Strength of the climate-HFRS association in 2010. (TIF)

S13 Fig Strength of the climate-HFRS association in 2011. (TIF)

S14 Fig Strength of the climate-HFRS association in 2012. (TIF)

S15 Fig Strength of the climate-HFRS association in 2013. (TIF)

S16 Fig Strength of the climate-HFRS association in 2014. (TIF)

S17 Fig Strength of the climate-HFRS association in 2015. (TIF)

S18 Fig Strength of the climate-HFRS association in 2016. (TIF)

S19 Fig Hotspot map of climate-HFRS association in 2005. (TIF)

S20 Fig Hotspot map of climate-HFRS association in 2006. (TIF)

S21 Fig Hotspot map of climate-HFRS association in 2007. (TIF)

S22 Fig Hotspot map of climate-HFRS association in 2008. (TIF)

S23 Fig Hotspot map of climate-HFRS association in 2009. (TIF)

S24 Fig Hotspot map of climate-HFRS association in 2010. (TIF)

S25 Fig Hotspot map of climate-HFRS association in 2011. (TIF)

S26 Fig Hotspot map of climate-HFRS association in 2012. (TIF)

S27 Fig Hotspot map of climate-HFRS association in 2013. (TIF)

S28 Fig Hotspot map of climate-HFRS association in 2014. (TIF)

S29 Fig Hotspot map of climate-HFRS association in 2015. (TIF)

S30 Fig Hotspot map of climate-HFRS association in 2016. (TIF)

S31 Fig Strength of the climate-HFRS association in various months during the period 2005–2016. (TIF)

S32 Fig Hotspot of the climate-HFRS association in various months during the period 2005–2016. (TIF)

S33 Fig Q-statistics and its significance of the monthly coherency maps during 2005–2016. (TIF)

S34 Fig Partial dependence of the other five geographic factors in GBM model (including spatial coordinates). (TIF)

S35 Fig Relative importance of various explanatory variables and the partial dependence of various geographic factors in GBM^#^ model (excluding spatial coordinates). (TIF)
