# Supplementary material for: Spatiotemporal variation of the association between climate dynamics and HFRS outbreaks in Eastern China during 2005-2016 and its geographic determinants
Source: PLoS Negl Trop Dis. 2018 Jun 6;12(6):e0006554. doi: 10.1371/journal.pntd.0006554 (PMC6005641; doi:10.1371/journal.pntd.0006554)
Supplement: S1 Table — (DOCX) [file pntd.0006554.s001.docx]

S1 Table Performance of the multiple linear regression model

| **Factors** | **Estimate** | **Std. Error** | **t value** | **Pr (>\|t\|)** |
| --- | --- | --- | --- | --- |
| Intercept | 7.674e-01 | 2.306e-03 | 332.819 | <2e-16*** |
| East coordinate | 3.932e-08 | 8.191e-10 | 48.004 | <2e-16*** |
| North coordinate | -3.696e-08 | 4.117e-10 | -89.768 | <2e-16*** |
| Elevation | 4.715e-06 | 6.762e-07 | 6.973 | 3.16e-12*** |
| Distance to coastline | 5.918e-08 | 8.762e-10 | 67.534 | <2e-16*** |
| Cropland | -1.200e-06 | 2.643e-07 | -4.542 | 5.60e-06*** |
| Woodland | 7.629e-07 | 2.636e-07 | 2.895 | 0.00380** |
| Grassland | 5.805e-06 | 3.493e-07 | 16.621 | <2e-16*** |
| Water | -1.864e-06 | 5.911e-07 | -3.154 | 0.00161** |
| Urban | 1.137e-05 | 6.865e-07 | 16.567 | <2e-16*** |
| Barren | 1.651e-05 | 6.414e-07 | 25.737 | <2e-16*** |

*** denotes that significant at 0.001 level, ** denotes that significant at 0.01 level.
