# Supplementary material for: Spatiotemporal variation of the association between climate dynamics and HFRS outbreaks in Eastern China during 2005-2016 and its geographic determinants
Source: PLoS Negl Trop Dis. 2018 Jun 6;12(6):e0006554. doi: 10.1371/journal.pntd.0006554 (PMC6005641; doi:10.1371/journal.pntd.0006554)
Supplement: S1 Text — (DOCX) [file pntd.0006554.s002.docx]

S1 Text The GBM^#^ model (excluding spatial coordinates)

The GBM^#^ model was used that excluded the spatial coordinates of the ten explanatory variables to avoid the interaction affected by spatial coordinates and explore some more detailed information about the geographic factors effects on climate-HFRS association. 10-fold cross validation analysis of the accuracy performance of the GBM^#^ model gave *R*^2^=0.82, $MAE=2.44\times10^{-4}$, $RMSE=1.56\times10^{-2}$. The relative importance of the eight explanatory variables and the partial dependencies of the geographic variables are plotted in S35 Fig. A graph similar to that obtained by the GBM model above was derived as regards the relative importance (ranking) of the geographic factors on the climate-HFRS association. In addition to the variable “distance to coastline” (with importance score 29.67%), the grassland and woodland were two other important explanatory variables of the climate-HFRS association (importance scores, 15.97% and 13.31%, respectively). Similar trends of the partial dependence for the same geographic factors can be found in Fig 4 and S35 Fig. Since in the GBM^#^ model the “distance to coastline” was the most important among the eight geographic factors, partial dependence of “distance to coastline” in S35 Fig fully reflects the effects of “distance to coastline” on the climate-HFRS association (i.e., the smaller the “distance to the coastline” is, the larger is its contribution on the climate-HFRS association; subsequently, the contribution becomes weaker with increasing distance displaying a wave-like shape). The partial dependence of grassland exhibits a rapid increase-decrease-stable trend as a function of the grassland area. The positive relationship between the climate-HFRS association and the woodland was found, etc.
