# Supplementary figures and images for: Spatiotemporal variation of the association between climate dynamics and HFRS outbreaks in Eastern China during 2005-2016 and its geographic determinants
Source: PLoS Negl Trop Dis. 2018 Jun 6;12(6):e0006554. doi: 10.1371/journal.pntd.0006554 (PMC6005641; doi:10.1371/journal.pntd.0006554)

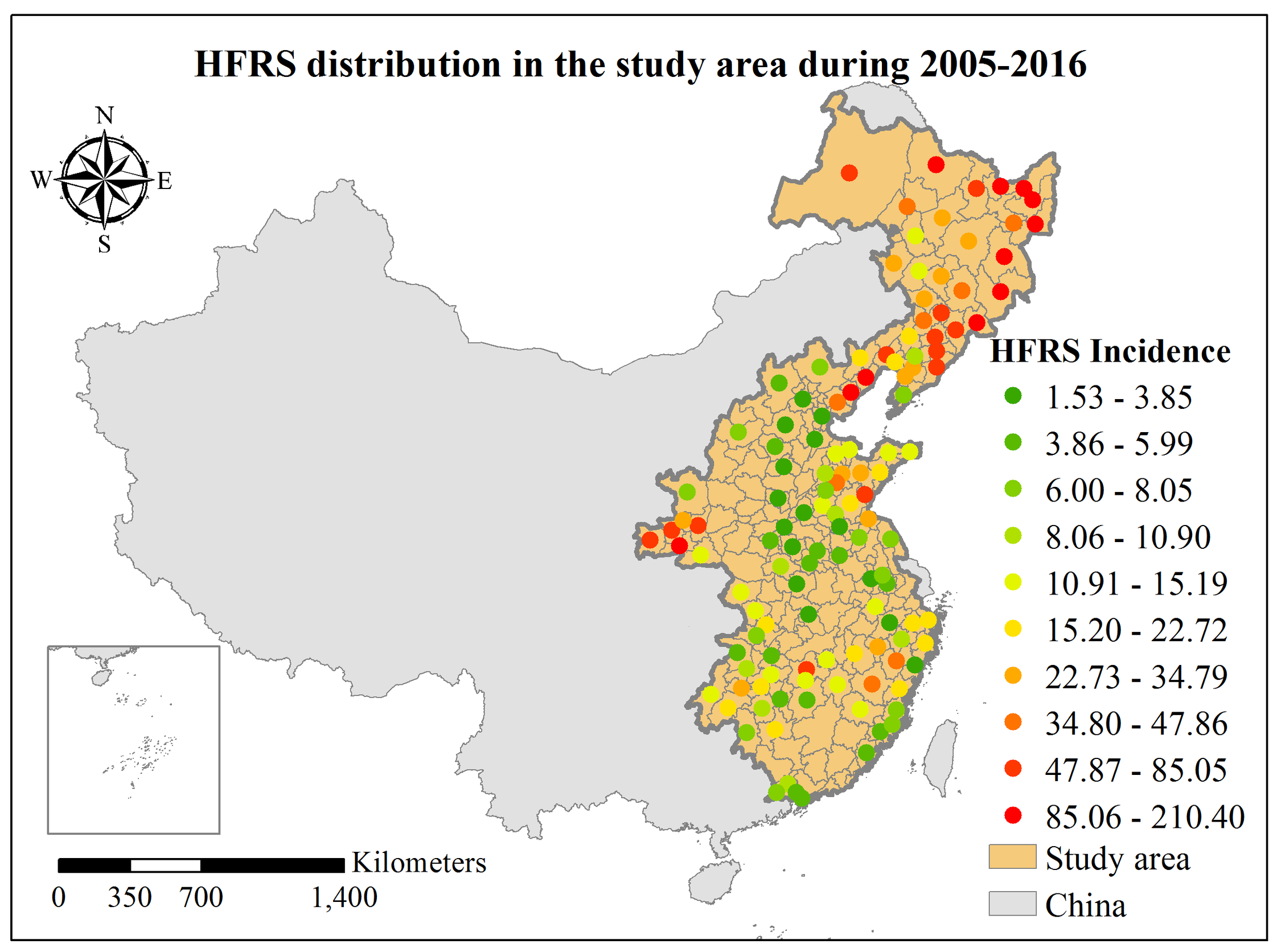

Supplement: S1 Fig — Population in 2015 was used to standardize the HFRS cases in each city. The unit of HFRS incidence is cases/100,000 population. (TIF) [file pntd.0006554.s003.tif]

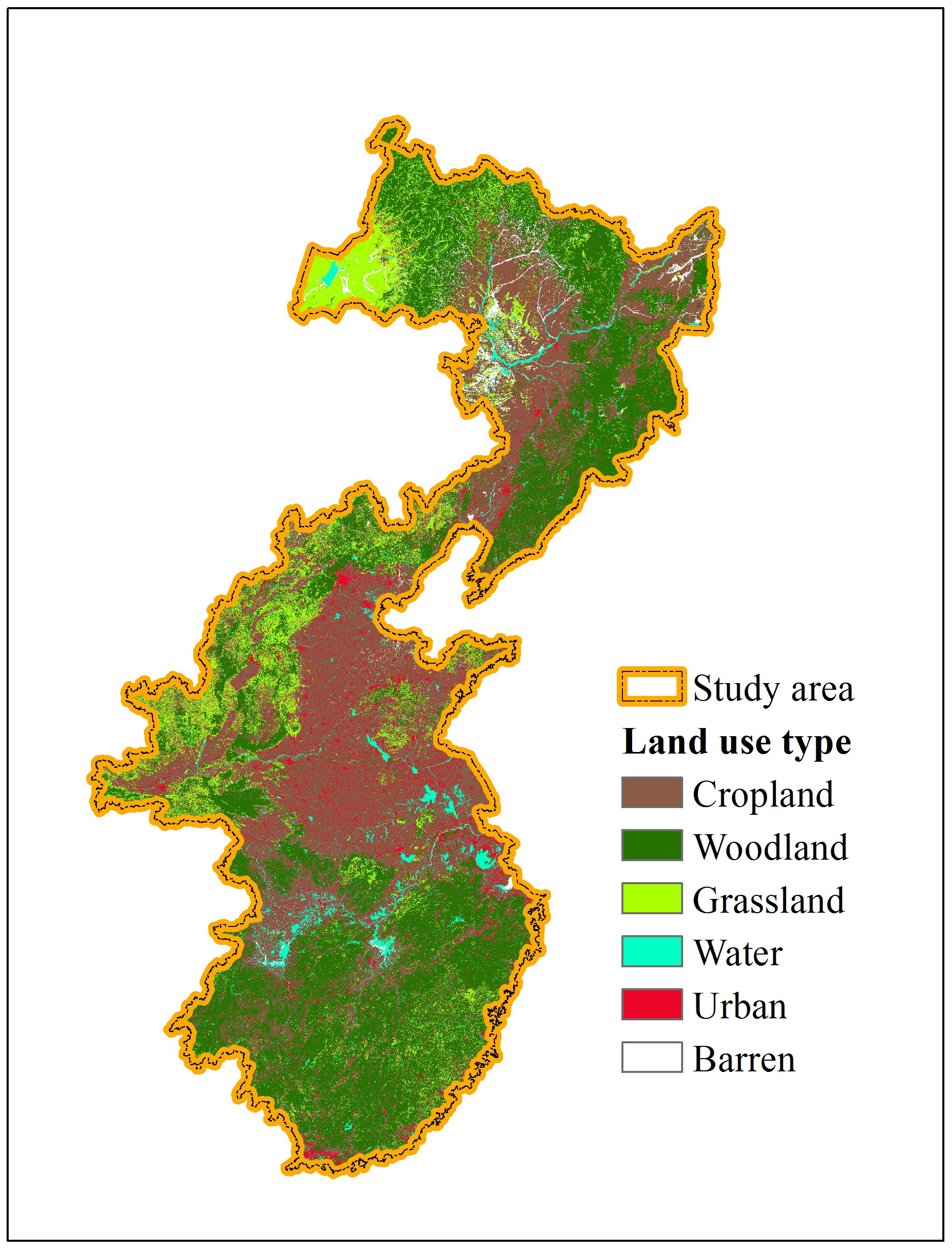

Supplement: S2 Fig — (TIF) [file pntd.0006554.s004.tif]

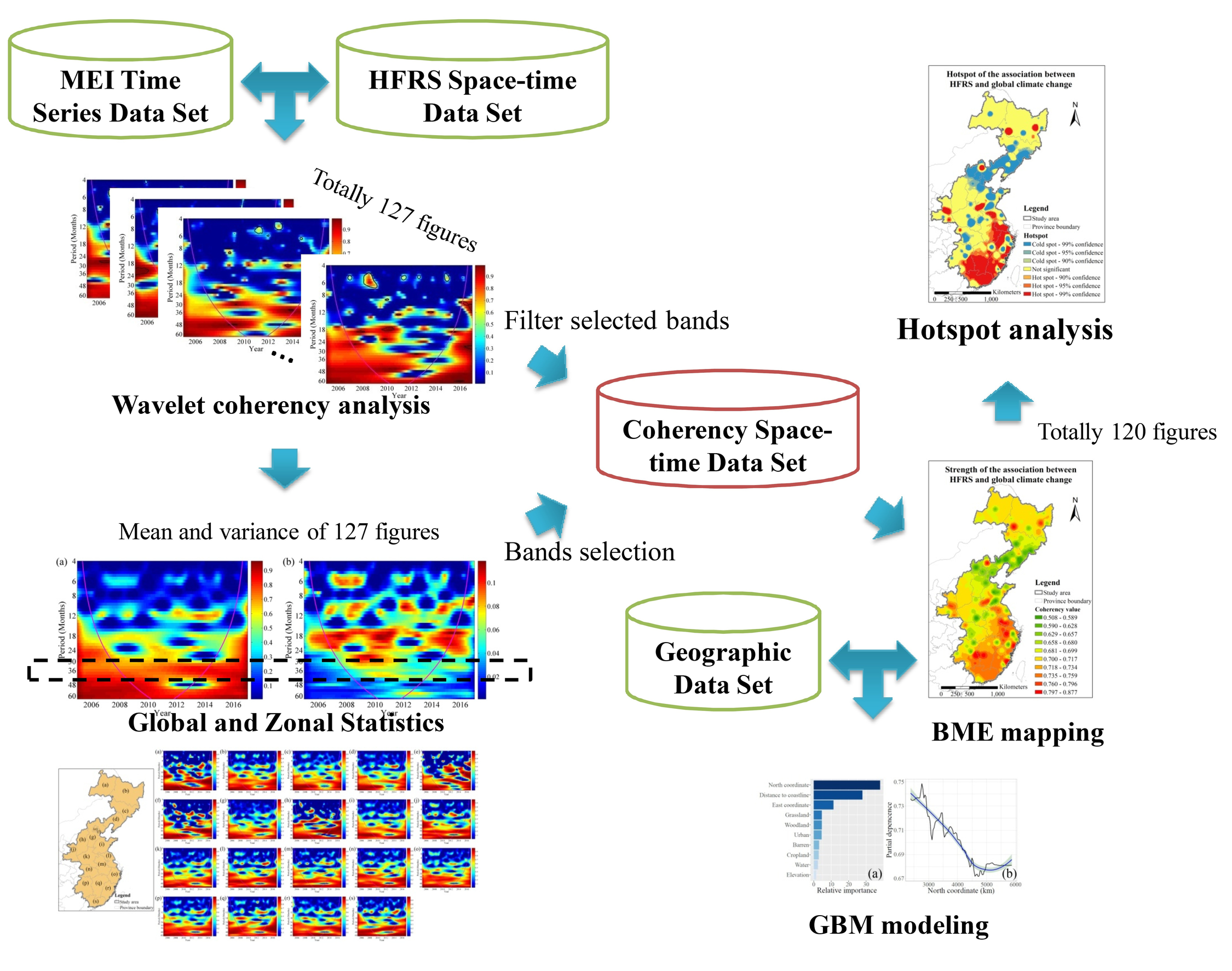

Supplement: S3 Fig — (TIF) [file pntd.0006554.s005.tif]

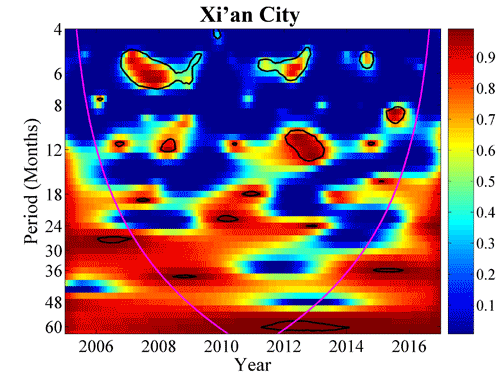

Supplement: S4 Fig — Purple line represents the cone of influence that delimits the region that is not influenced by edge effects; black line shows a = 5% significance level computed based on 500 bootstrap. (GIF) [file pntd.0006554.s006.gif]

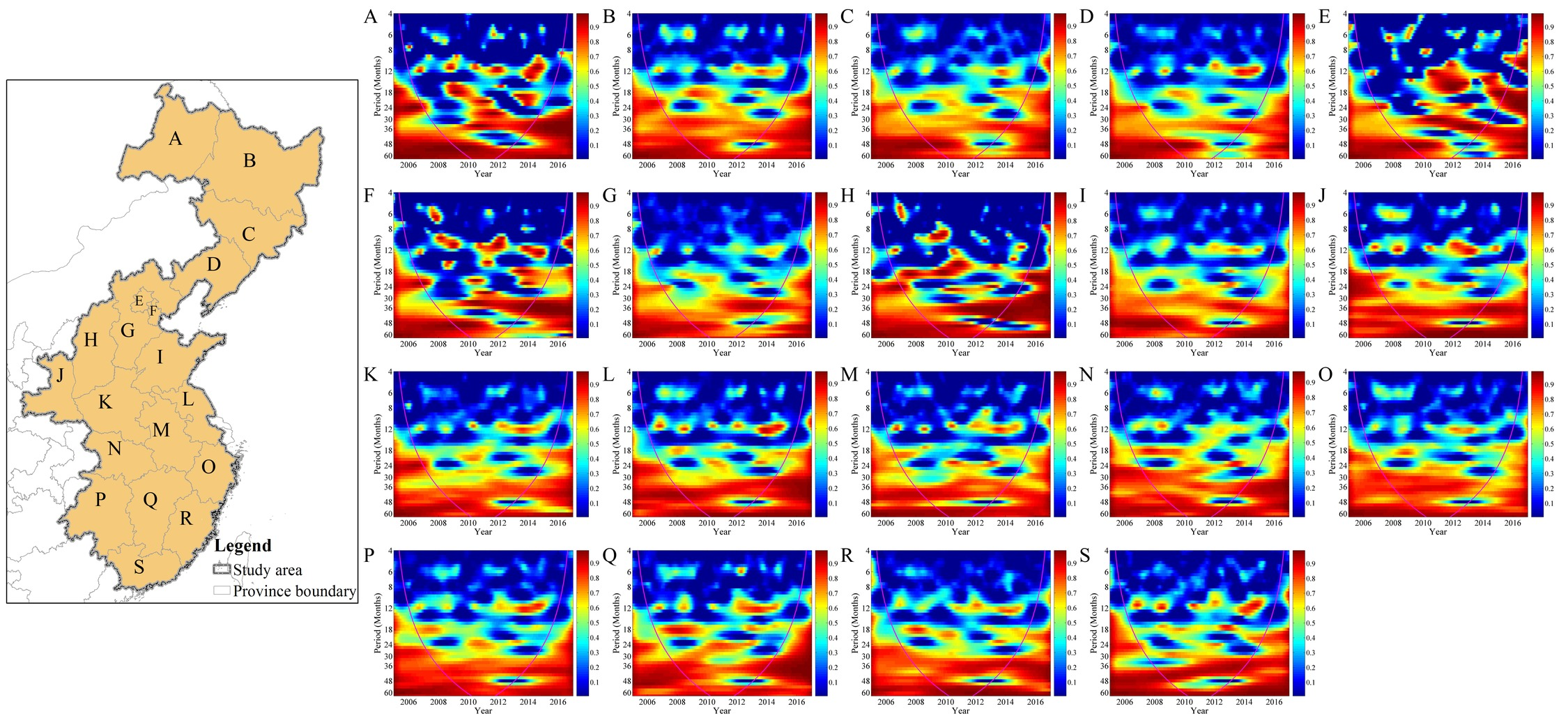

Supplement: S5 Fig — A–S represent Inner Mongolia, Heilongjiang, Jilin, Liaoning, Beijing, Tianjin, Hebei, Shanxi, Shandong, Shaanxi, Henan, Jiangsu, Anhui, Hubei, Zhejiang, Hunan, Jiangxi, Fujian and Guangdong, respectively. (TIF) [file pntd.0006554.s007.tif]

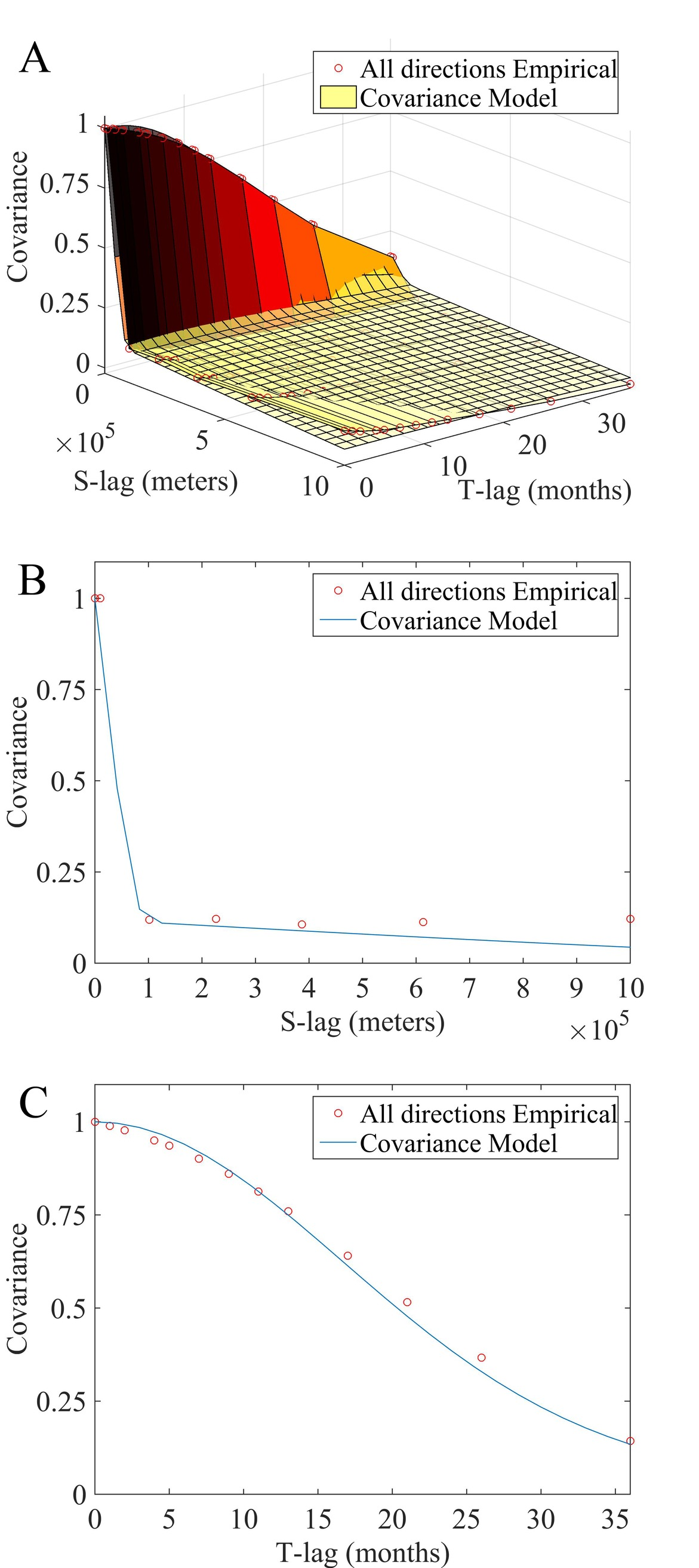

Supplement: S6 Fig — A Composite space-time empirical and fitted theoretical covariance; B empirical and fitted theoretical covariance when T-lag equals to 0; C empirical and fitted theoretical covariance when S-lag equals to 0. (TIF) [file pntd.0006554.s008.tif]

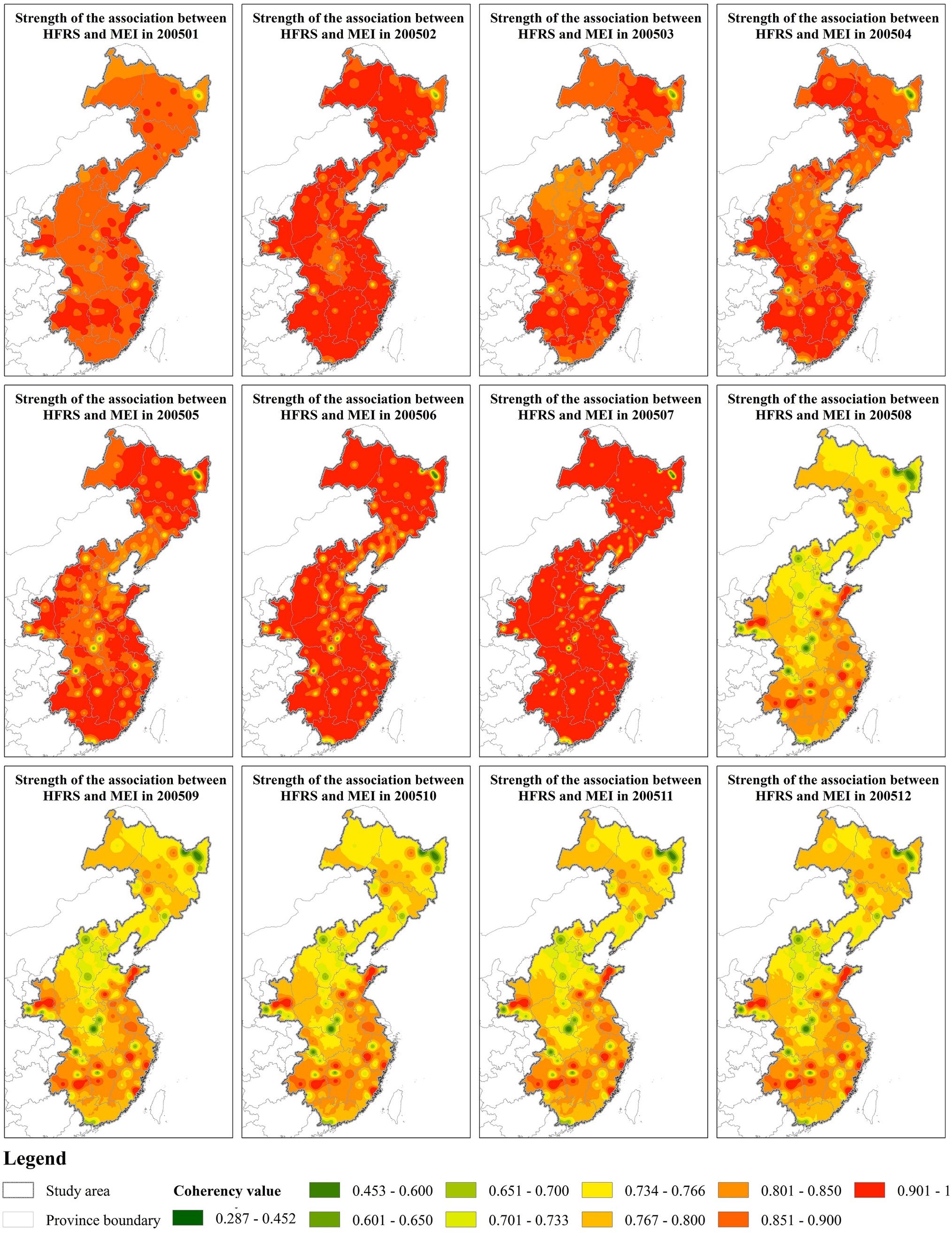

Supplement: S7 Fig — (TIF) [file pntd.0006554.s009.tif]

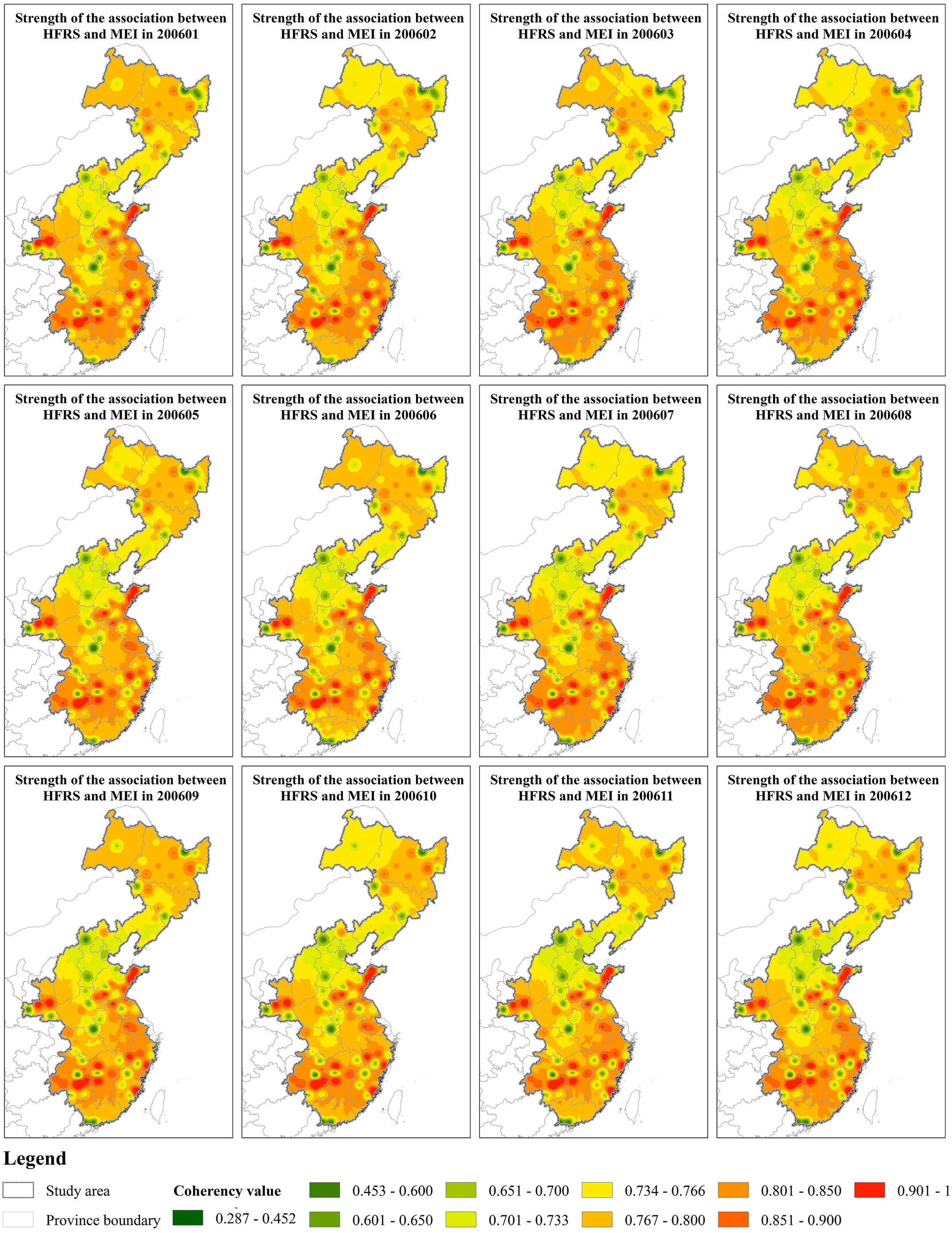

Supplement: S8 Fig — (TIF) [file pntd.0006554.s010.tif]

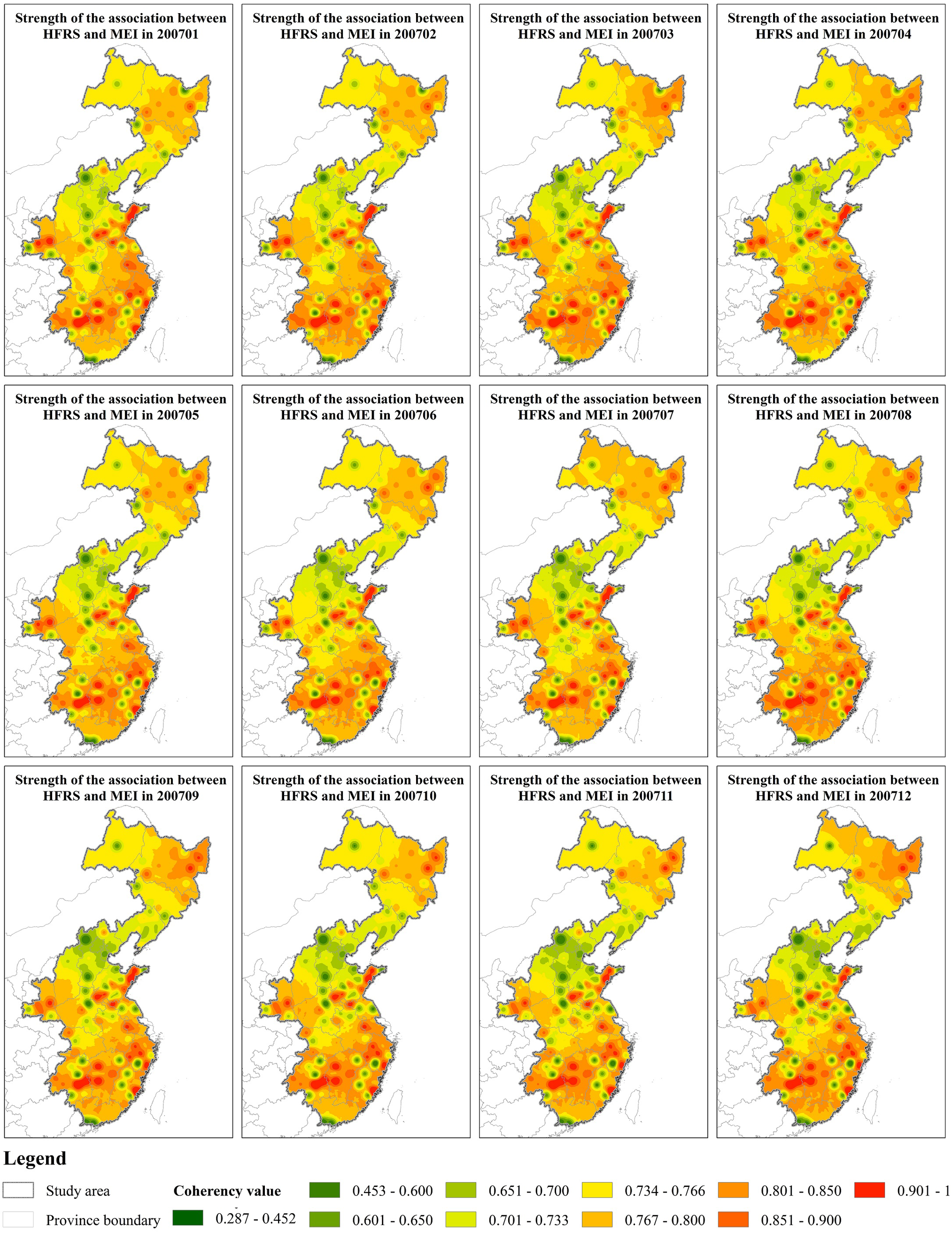

Supplement: S9 Fig — (TIF) [file pntd.0006554.s011.tif]

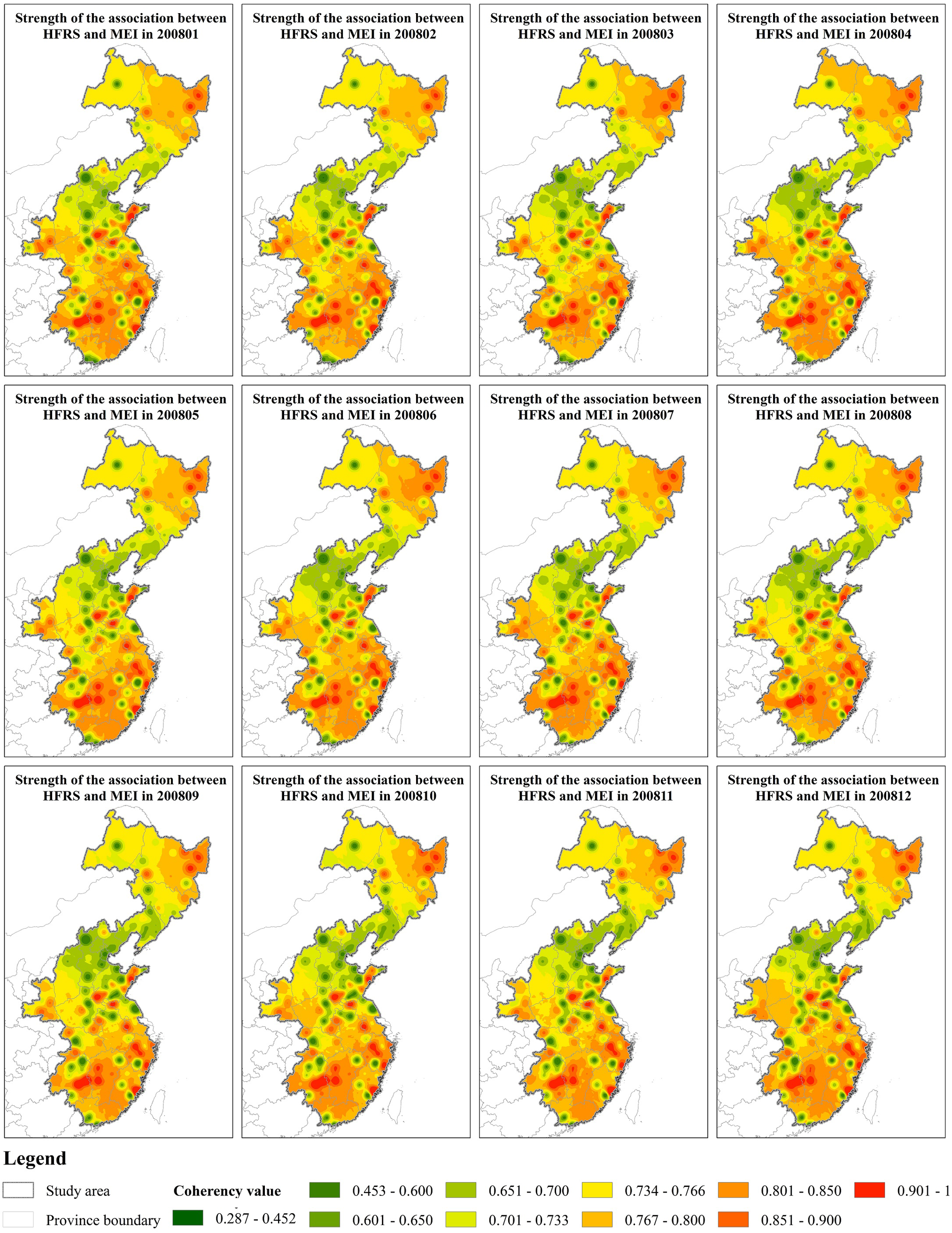

Supplement: S10 Fig — (TIF) [file pntd.0006554.s012.tif]

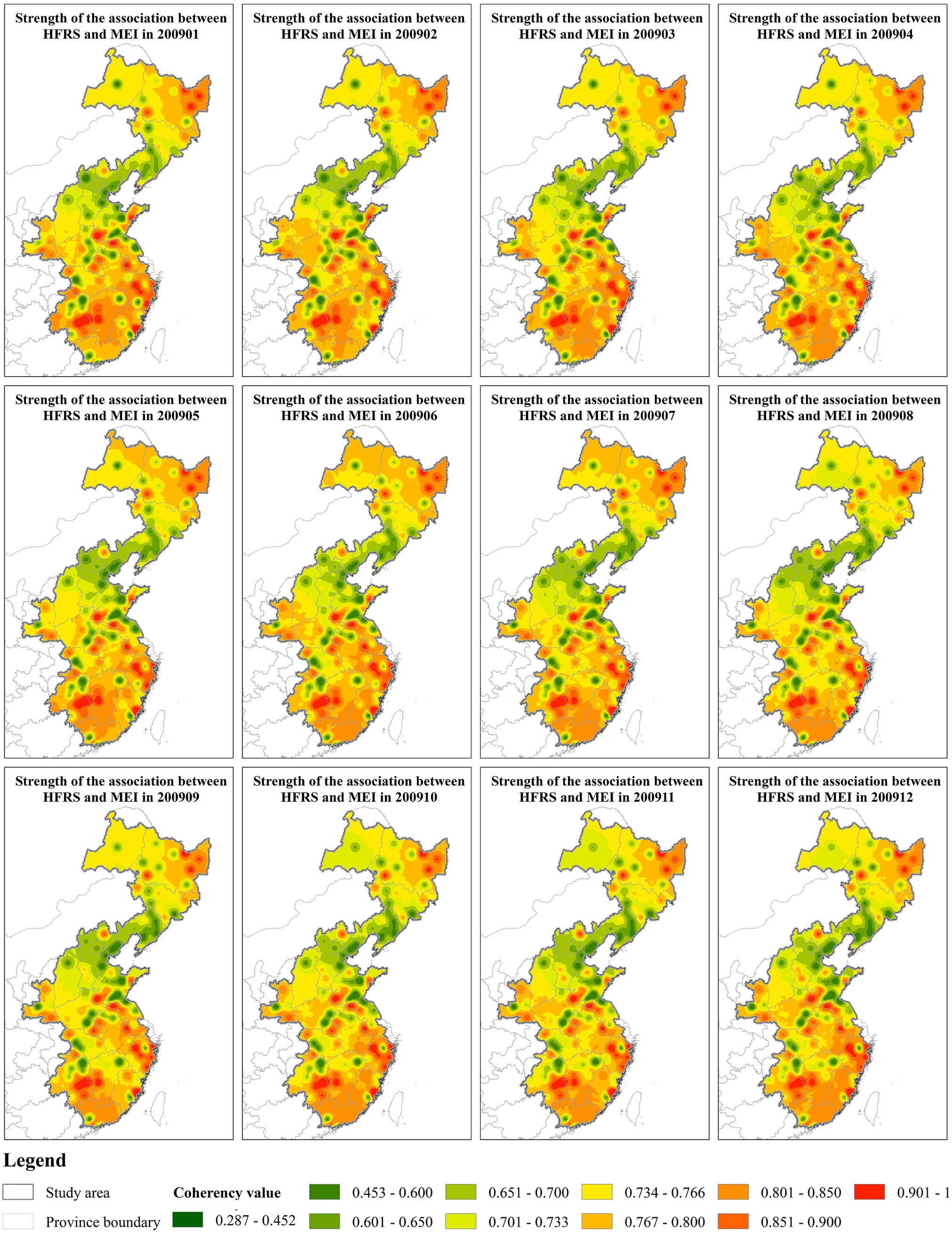

Supplement: S11 Fig — (TIF) [file pntd.0006554.s013.tif]

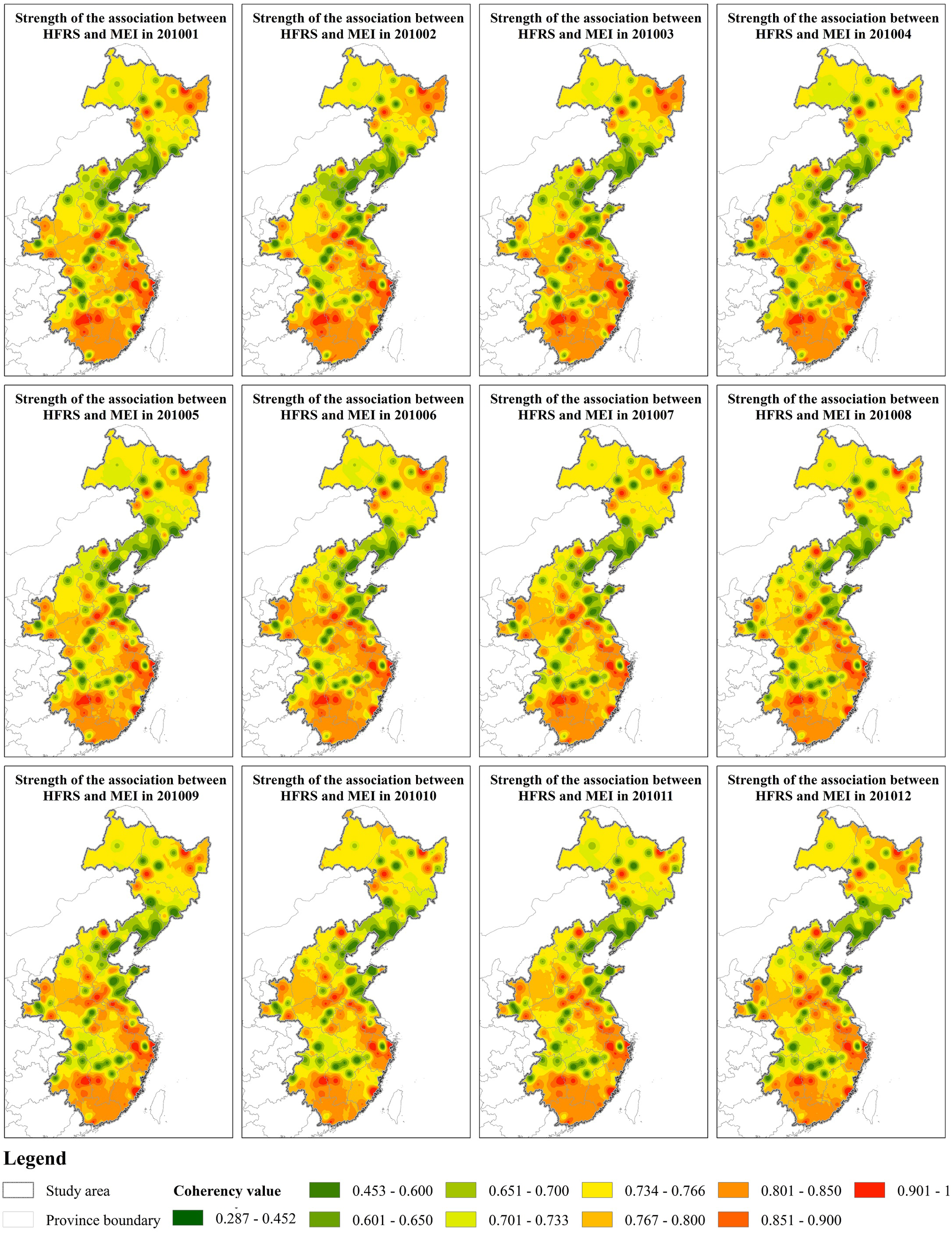

Supplement: S12 Fig — (TIF) [file pntd.0006554.s014.tif]

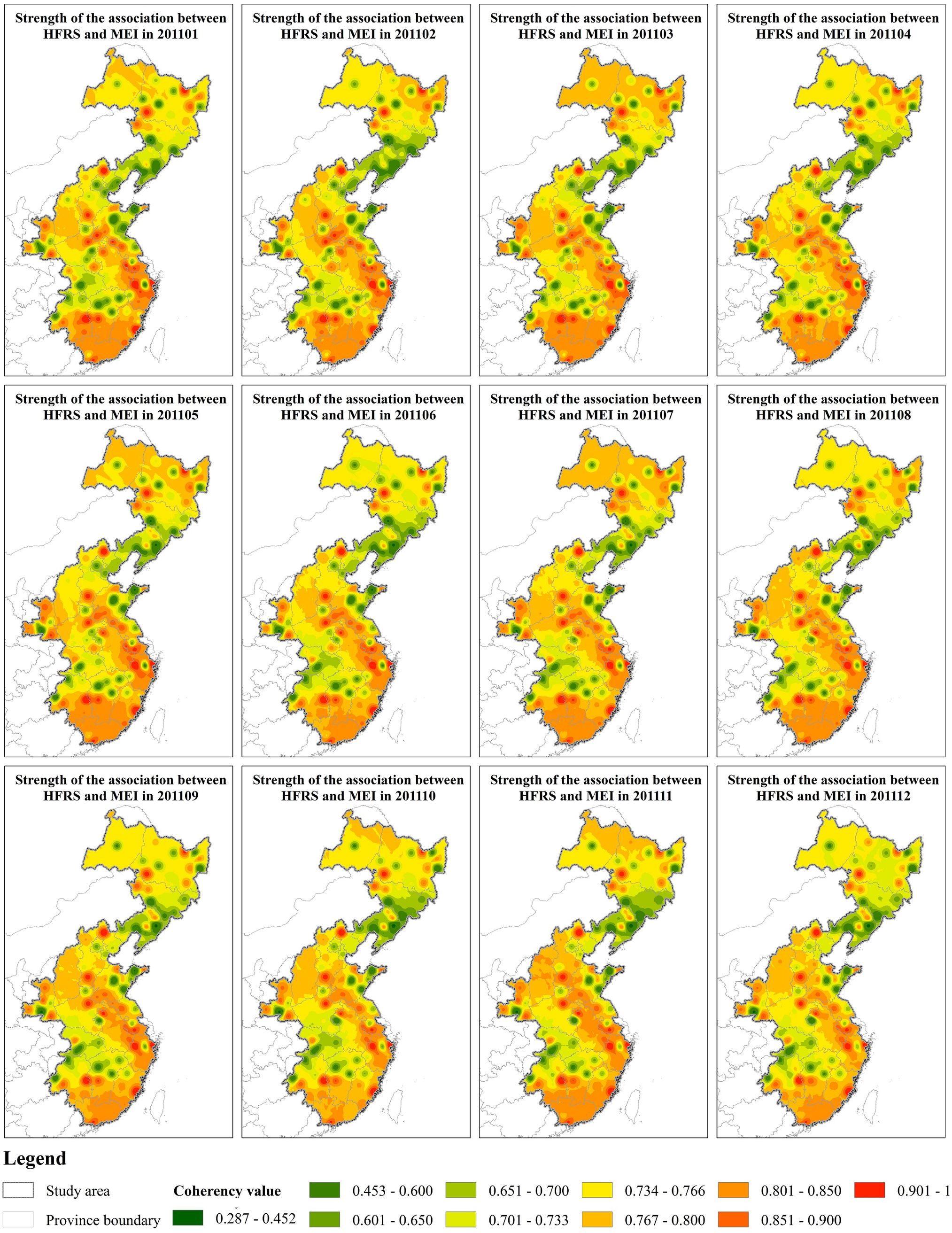

Supplement: S13 Fig — (TIF) [file pntd.0006554.s015.tif]

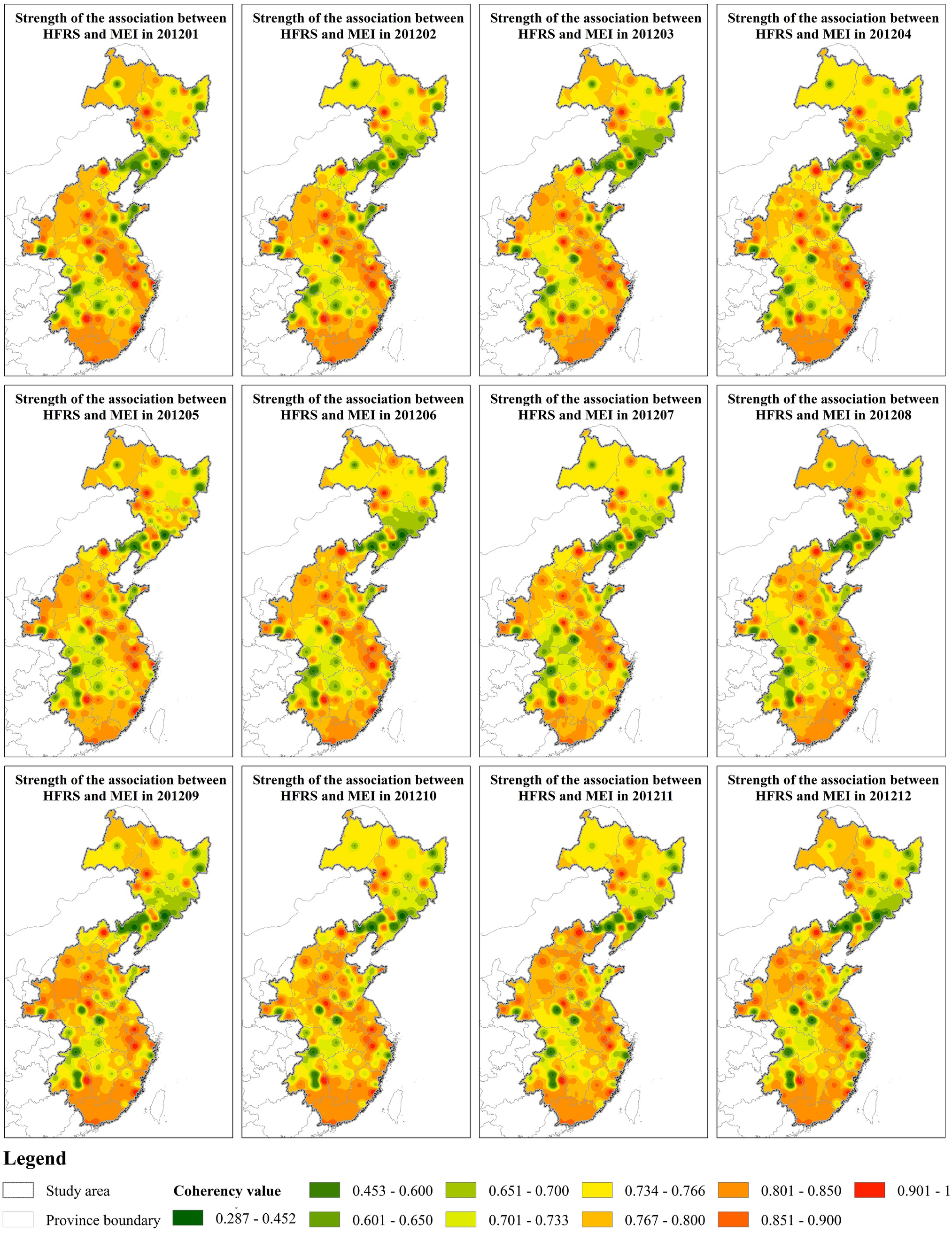

Supplement: S14 Fig — (TIF) [file pntd.0006554.s016.tif]

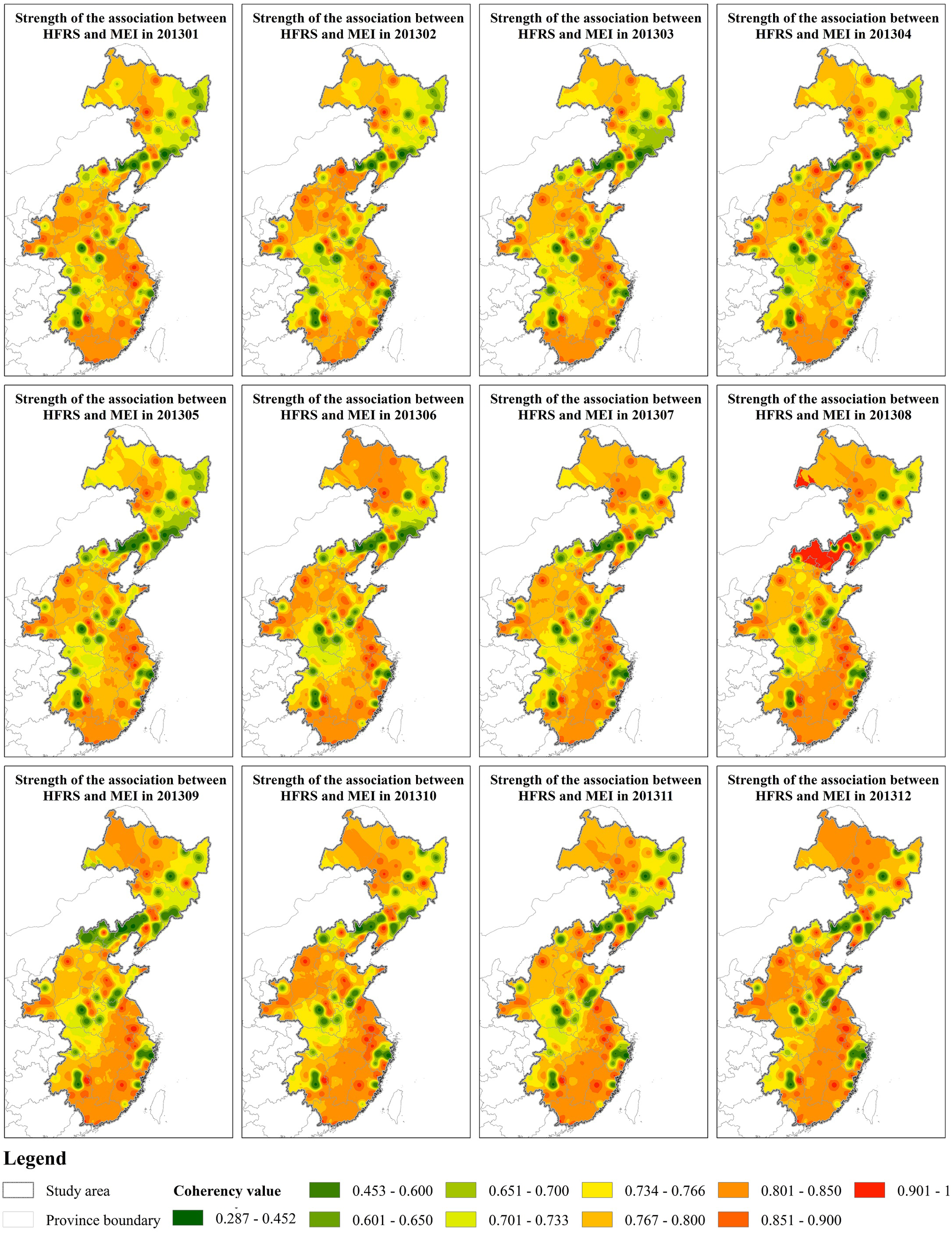

Supplement: S15 Fig — (TIF) [file pntd.0006554.s017.tif]

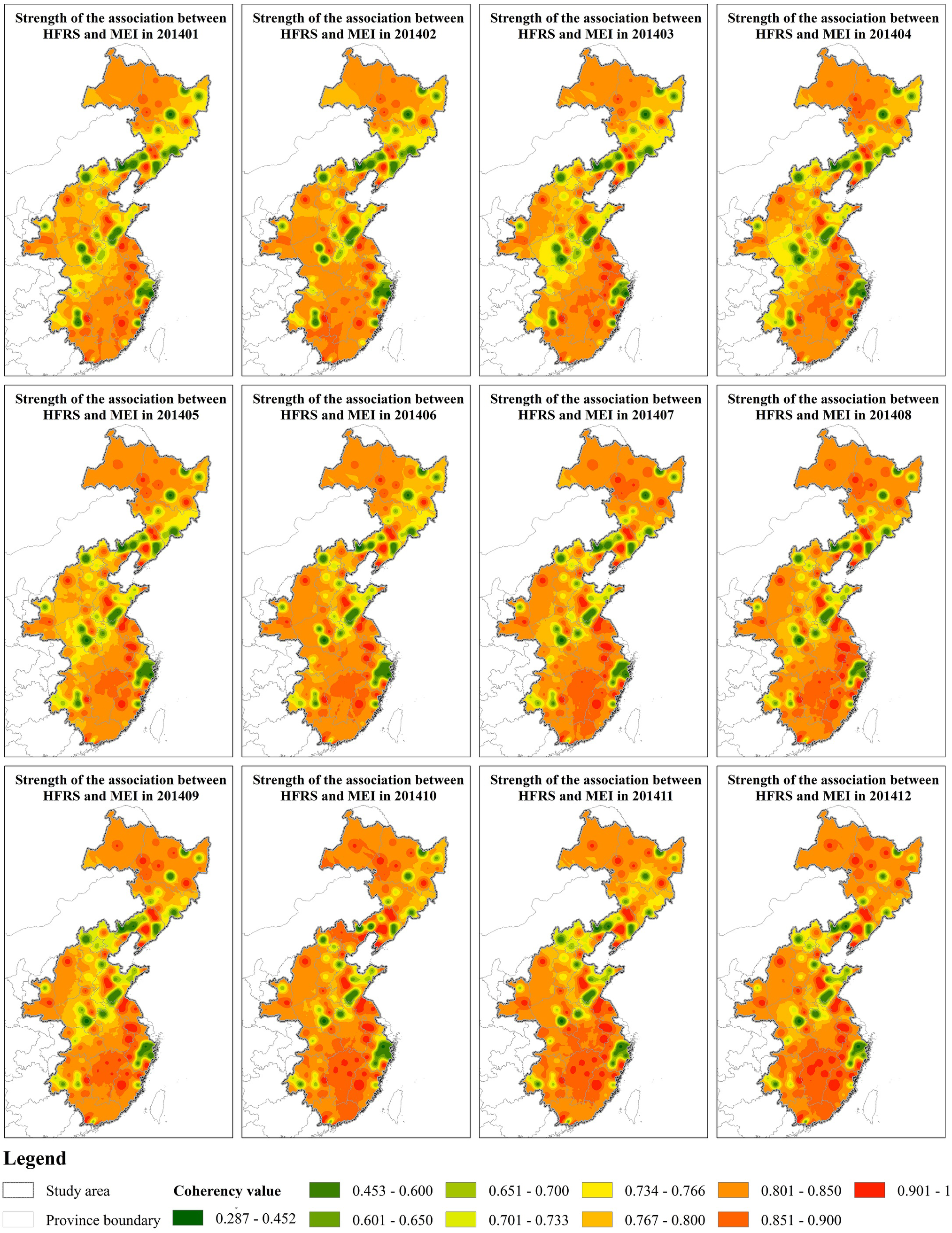

Supplement: S16 Fig — (TIF) [file pntd.0006554.s018.tif]

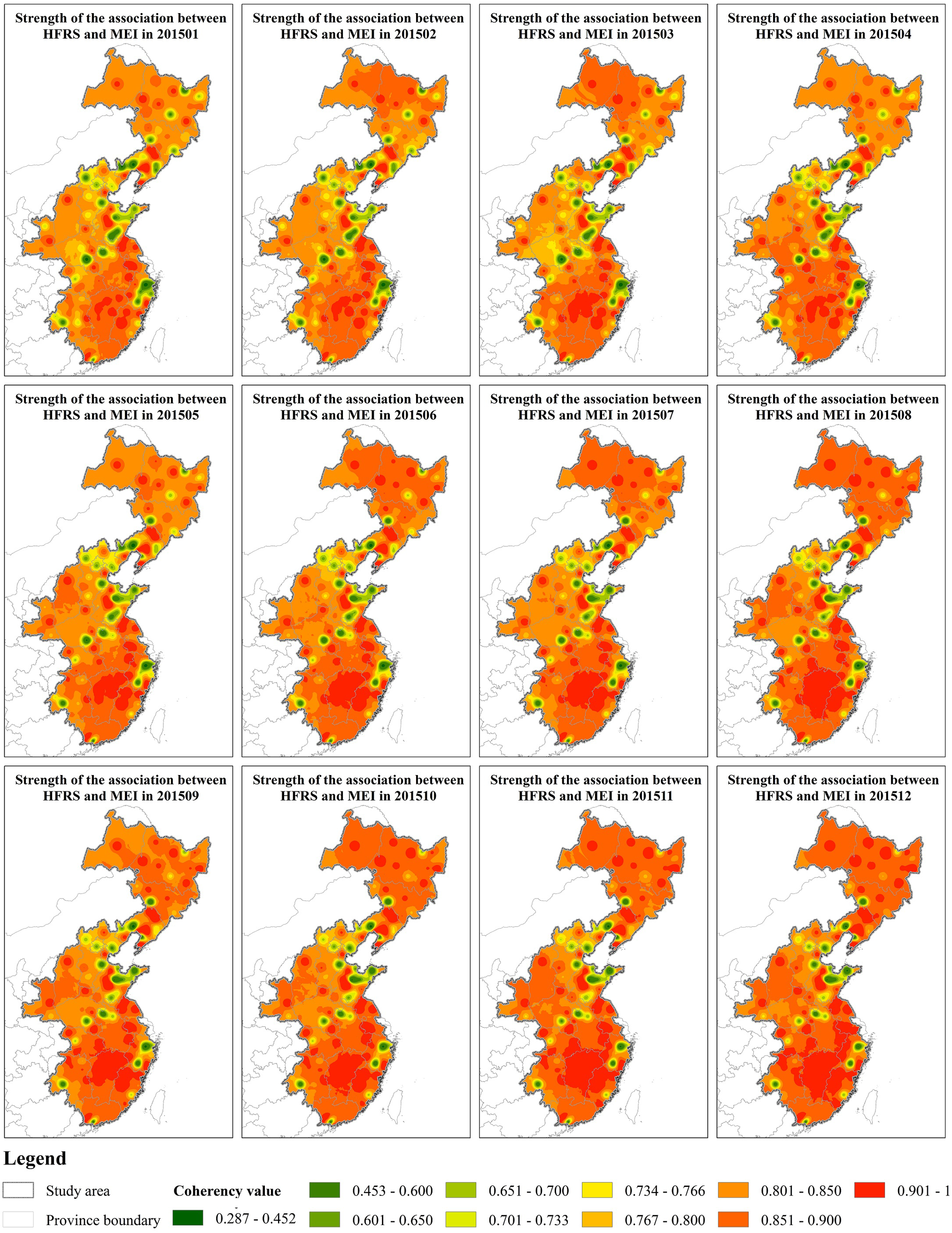

Supplement: S17 Fig — (TIF) [file pntd.0006554.s019.tif]

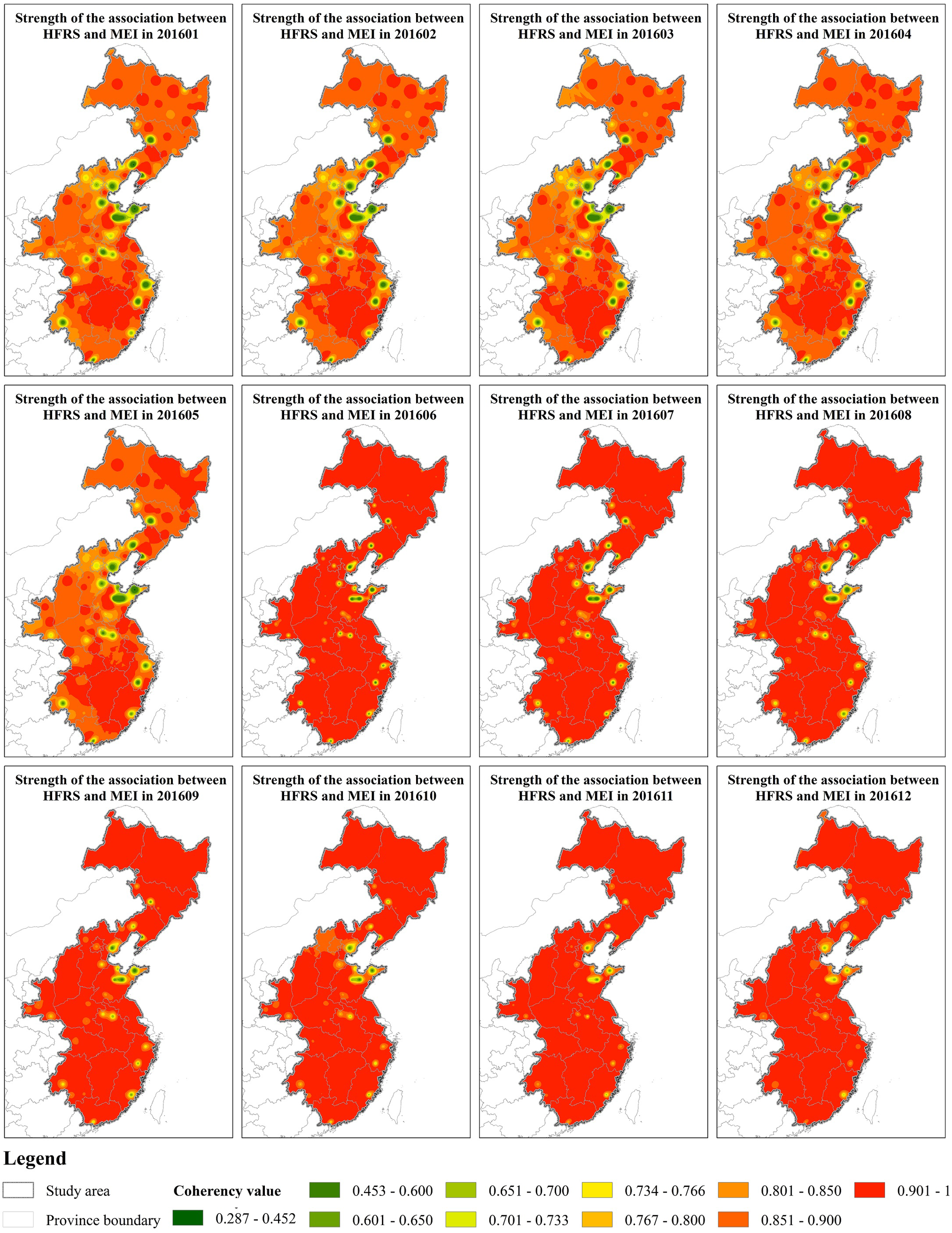

Supplement: S18 Fig — (TIF) [file pntd.0006554.s020.tif]

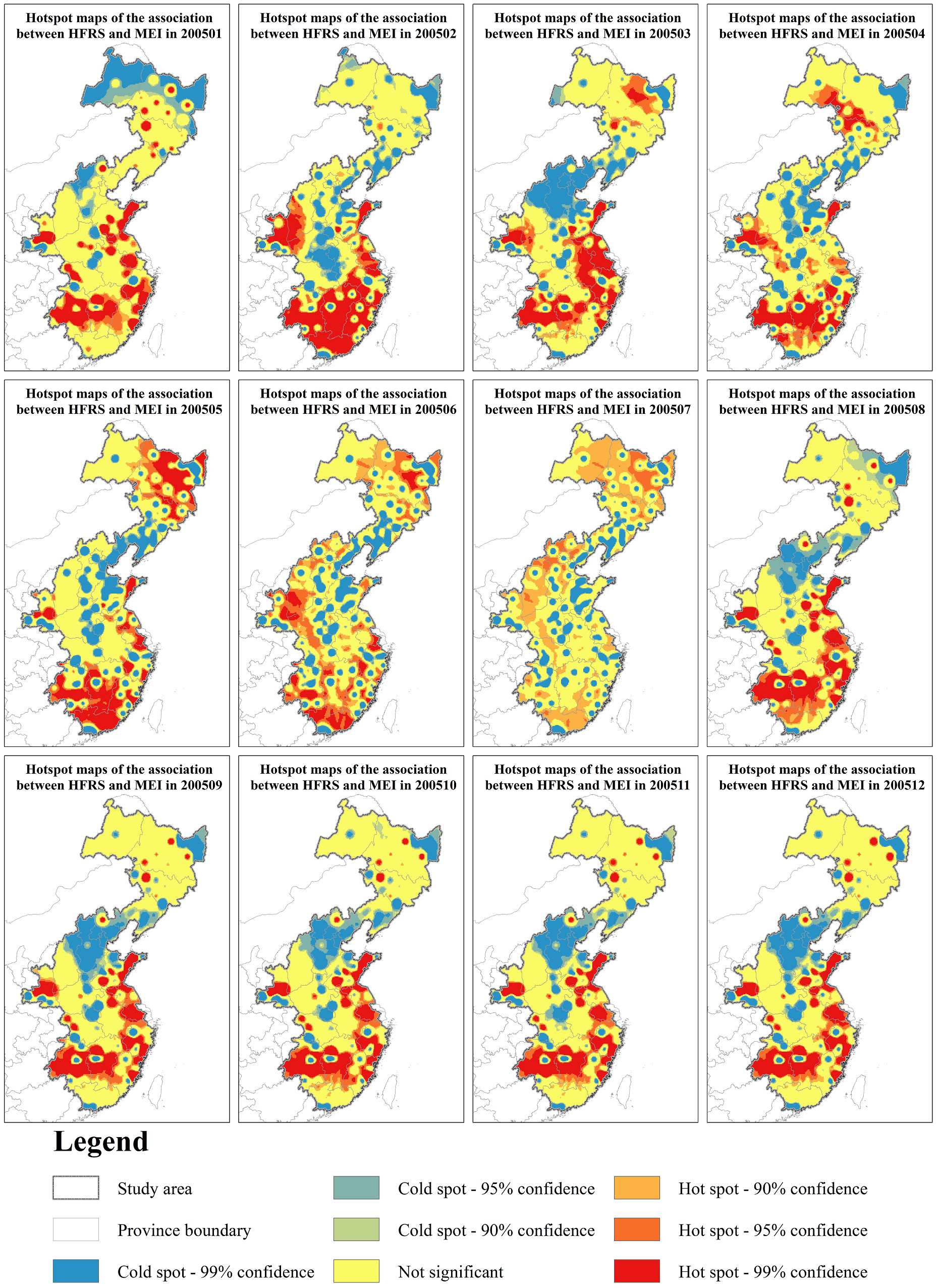

Supplement: S19 Fig — (TIF) [file pntd.0006554.s021.tif]

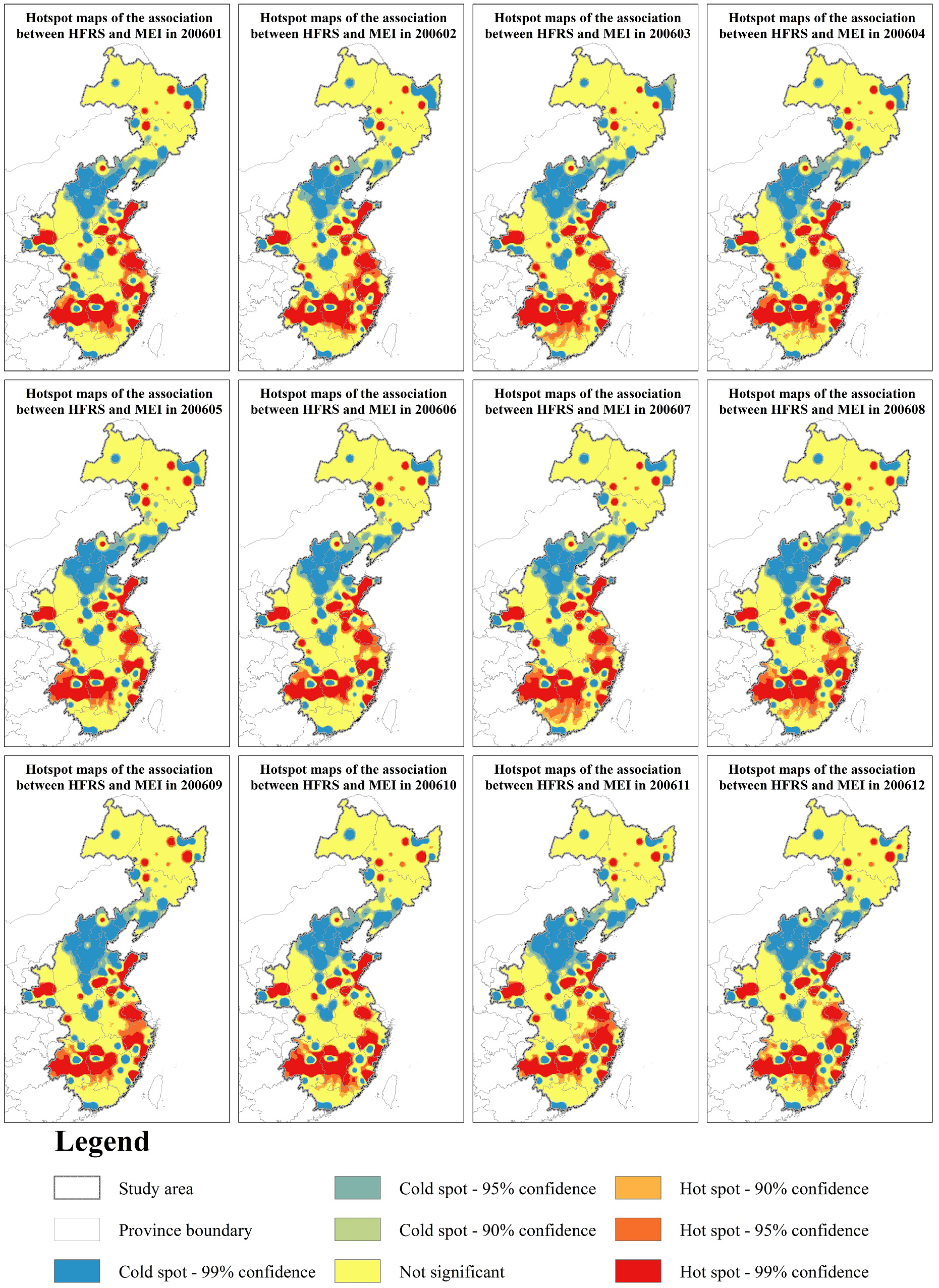

Supplement: S20 Fig — (TIF) [file pntd.0006554.s022.tif]

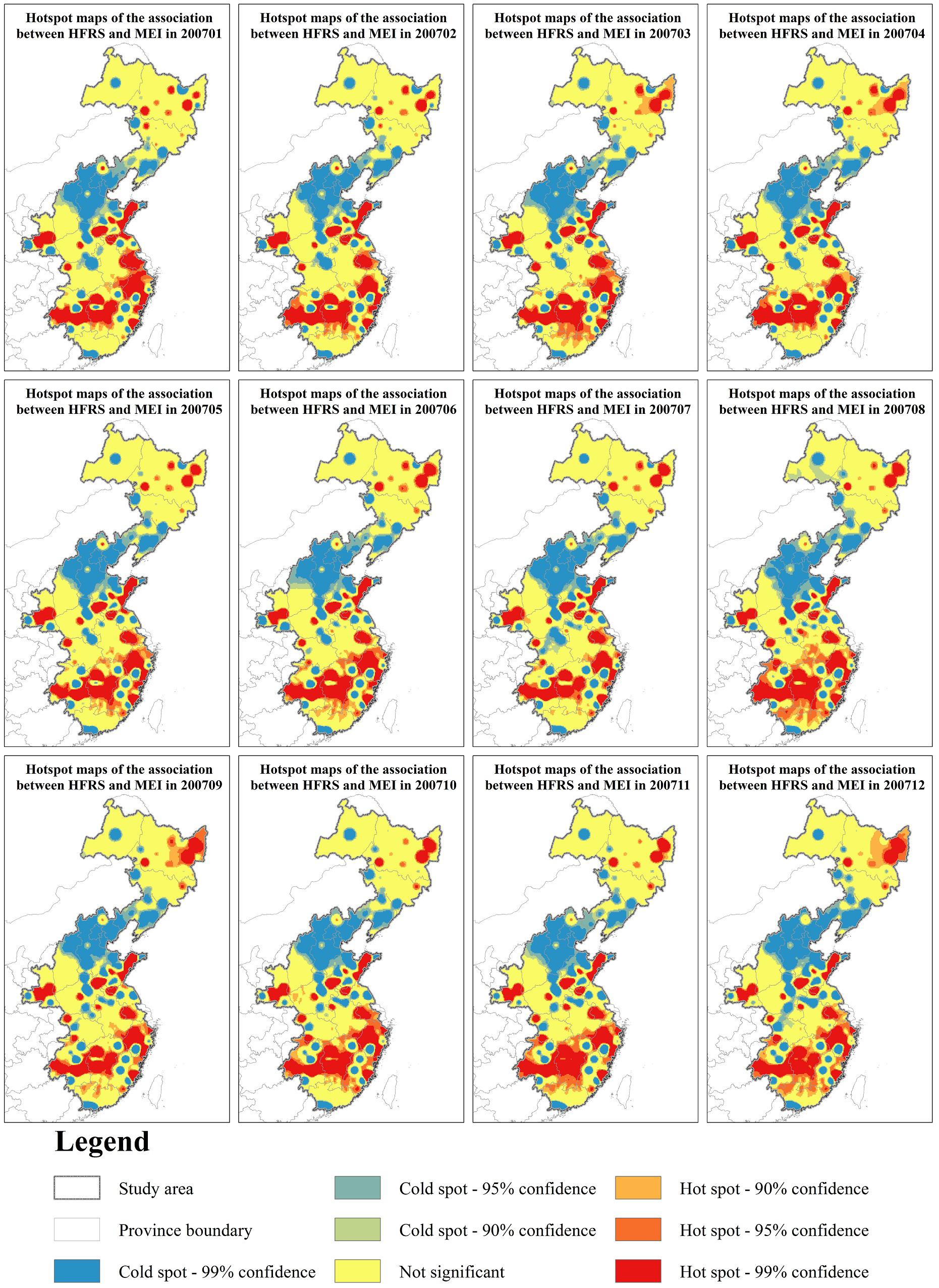

Supplement: S21 Fig — (TIF) [file pntd.0006554.s023.tif]

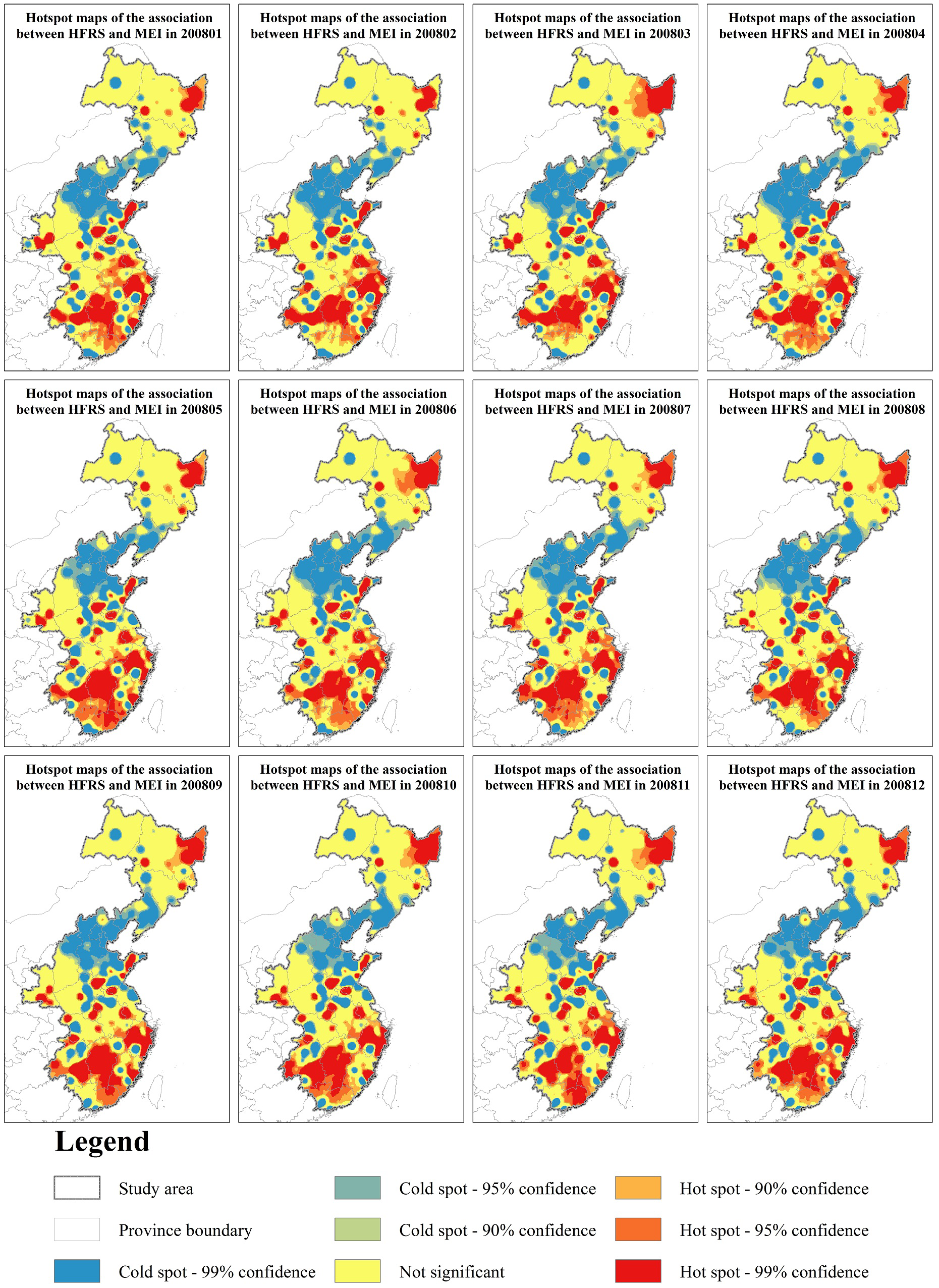

Supplement: S22 Fig — (TIF) [file pntd.0006554.s024.tif]

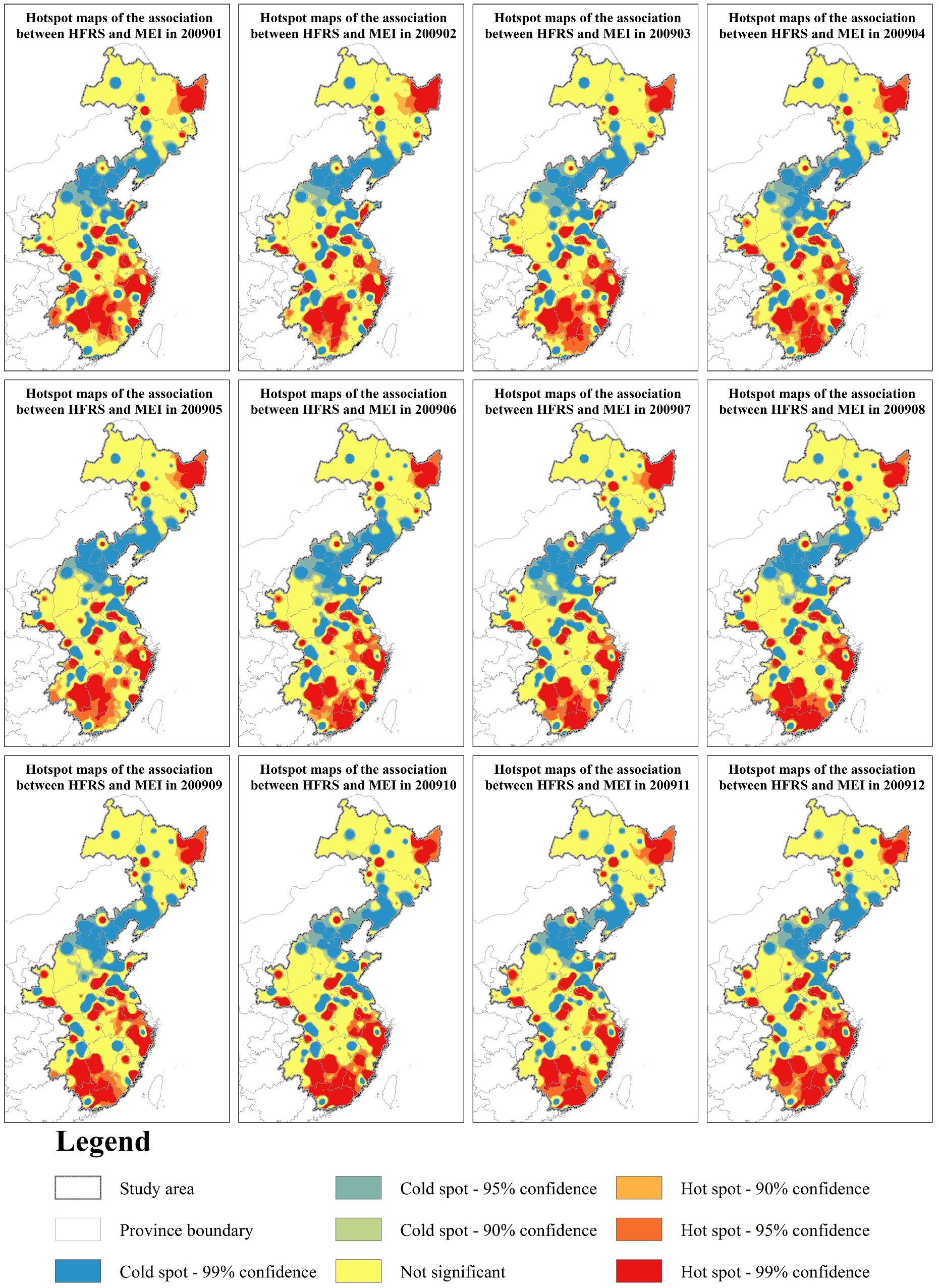

Supplement: S23 Fig — (TIF) [file pntd.0006554.s025.tif]

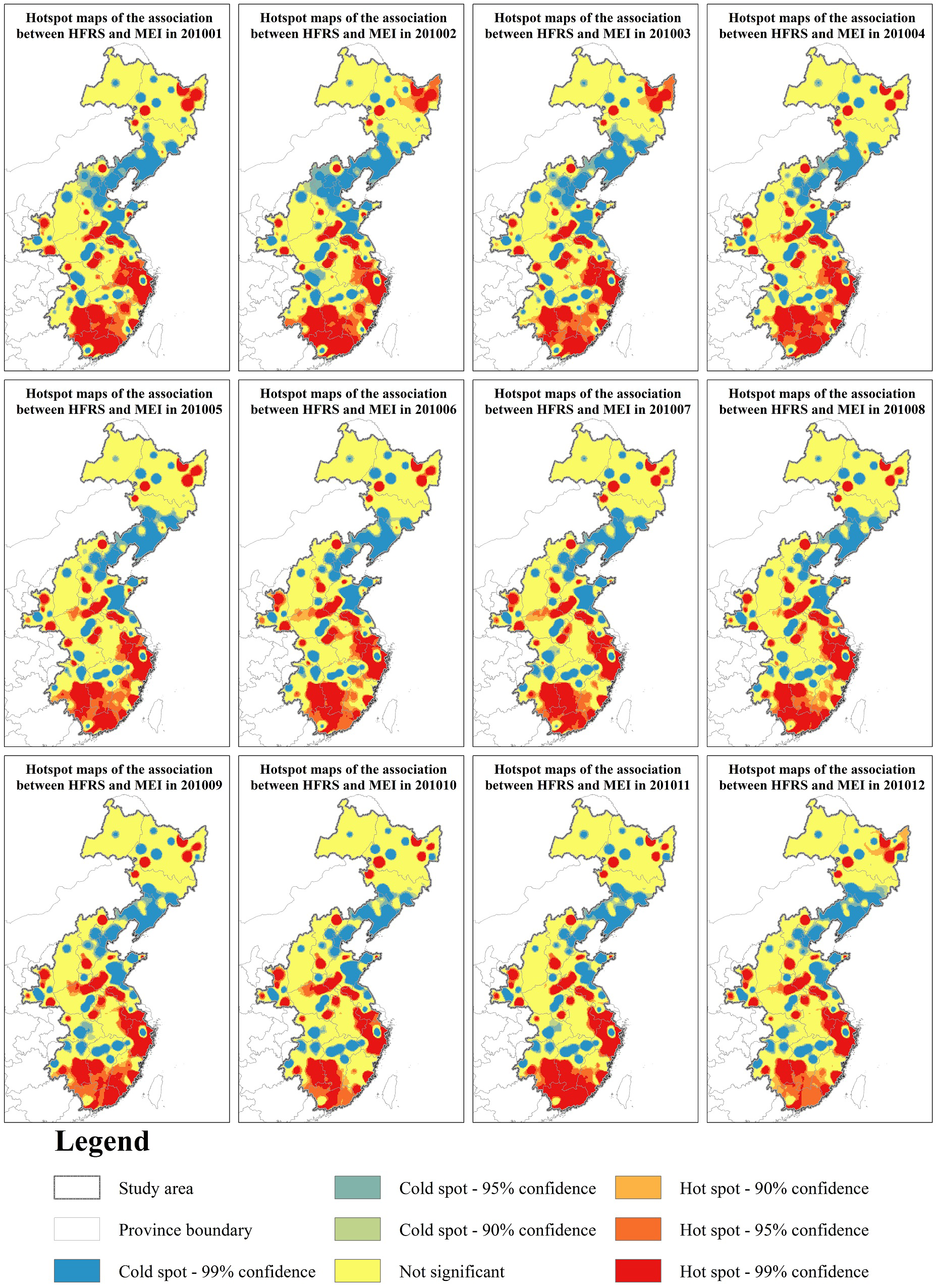

Supplement: S24 Fig — (TIF) [file pntd.0006554.s026.tif]

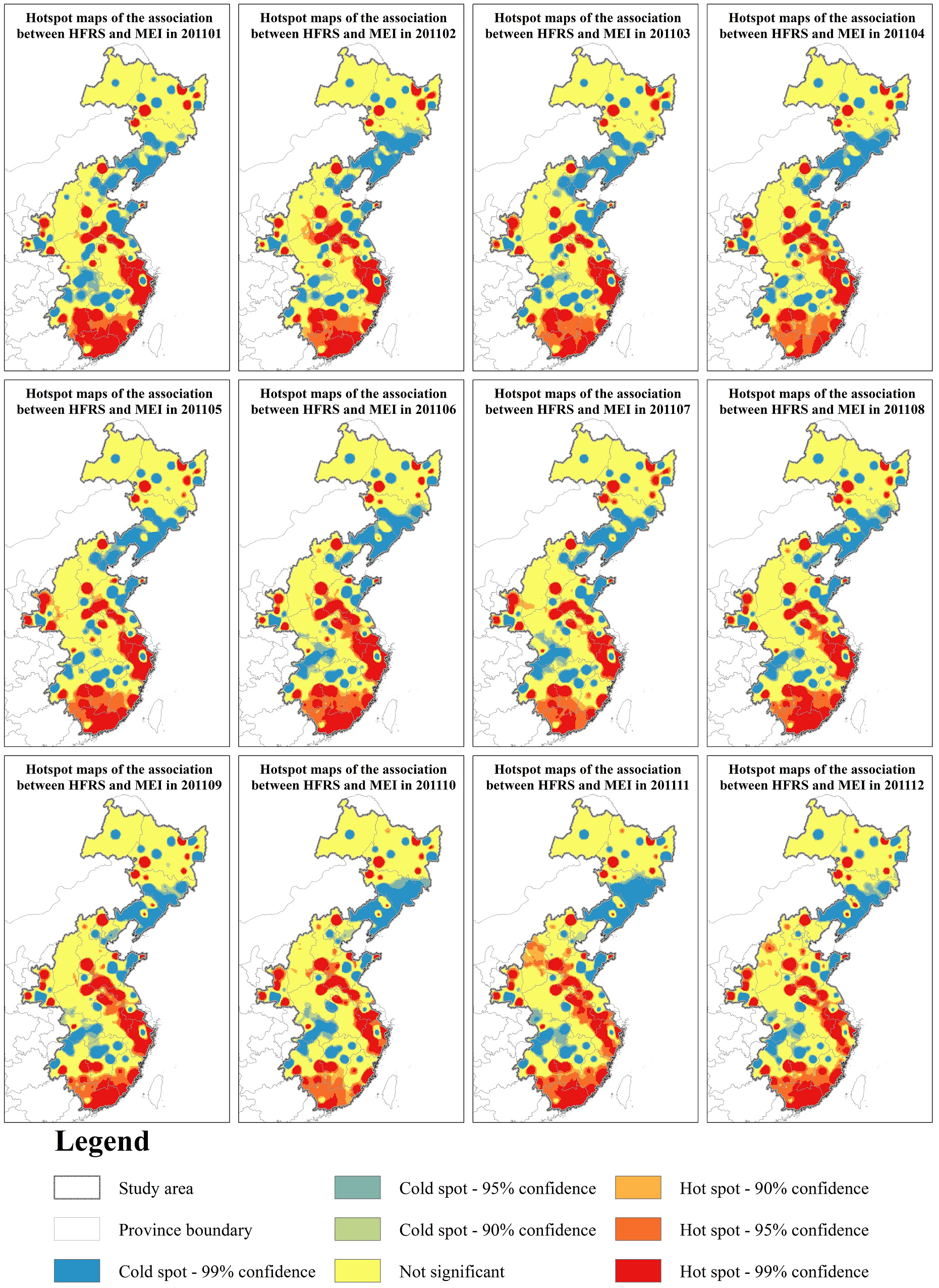

Supplement: S25 Fig — (TIF) [file pntd.0006554.s027.tif]

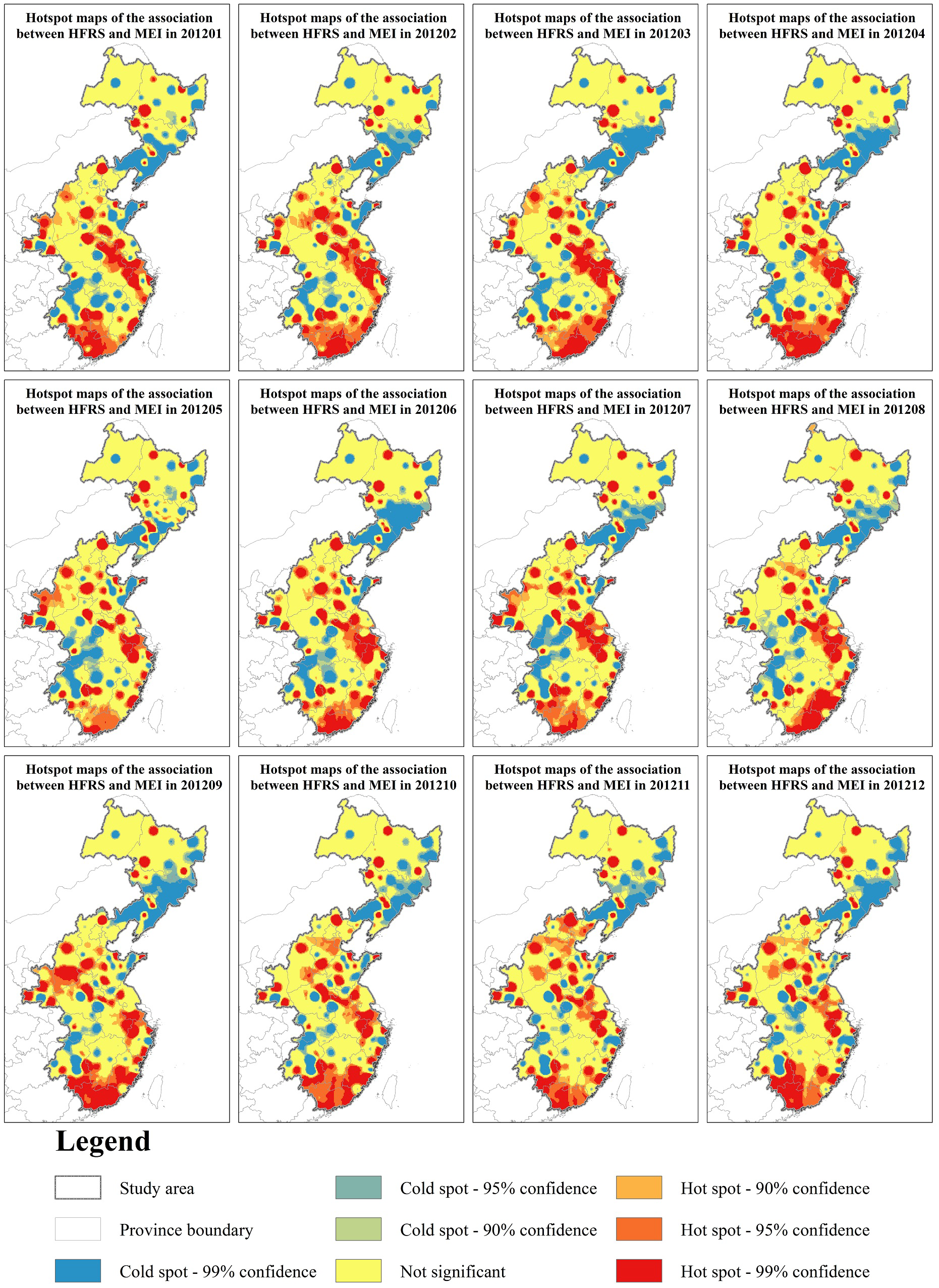

Supplement: S26 Fig — (TIF) [file pntd.0006554.s028.tif]

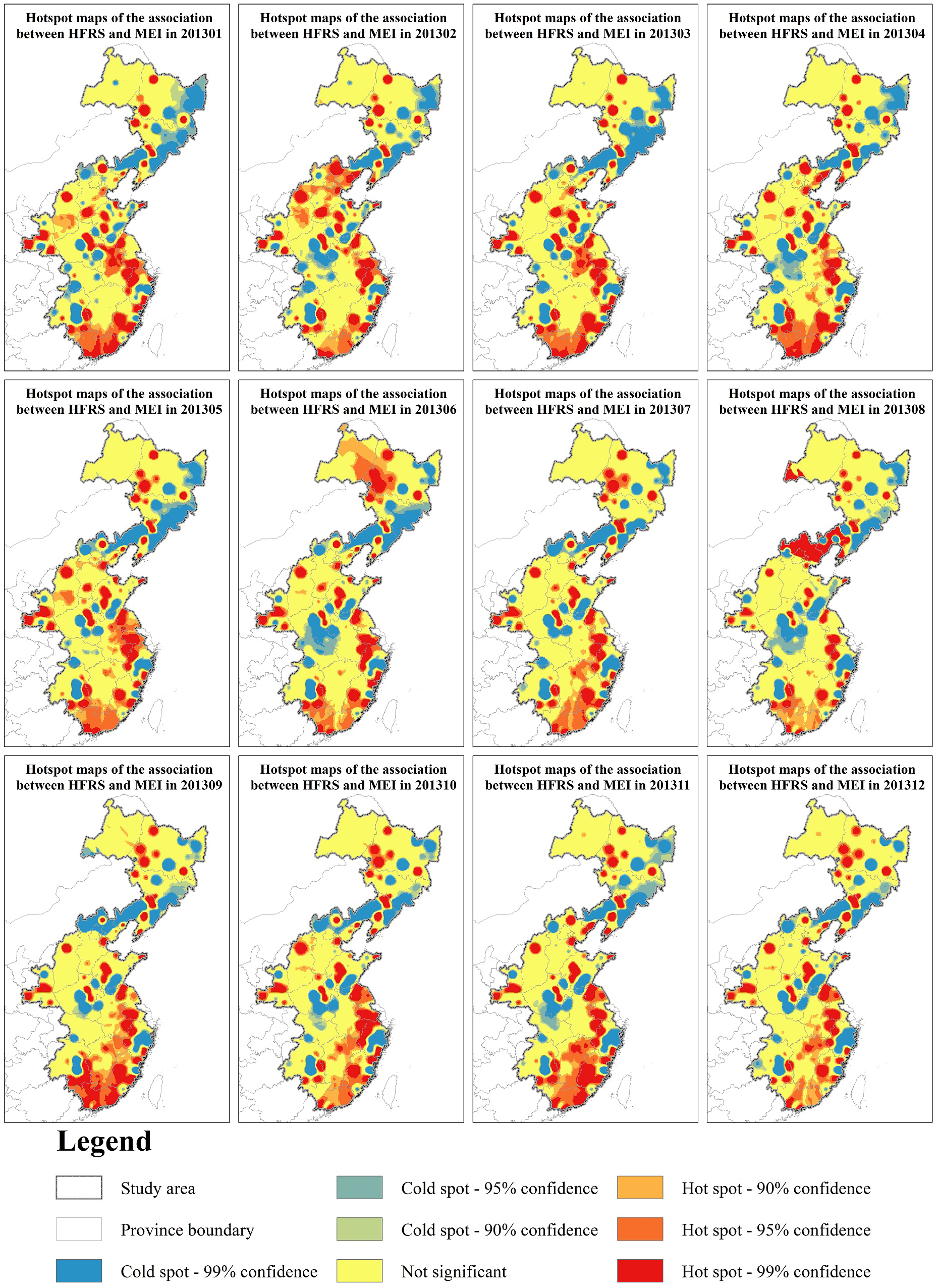

Supplement: S27 Fig — (TIF) [file pntd.0006554.s029.tif]

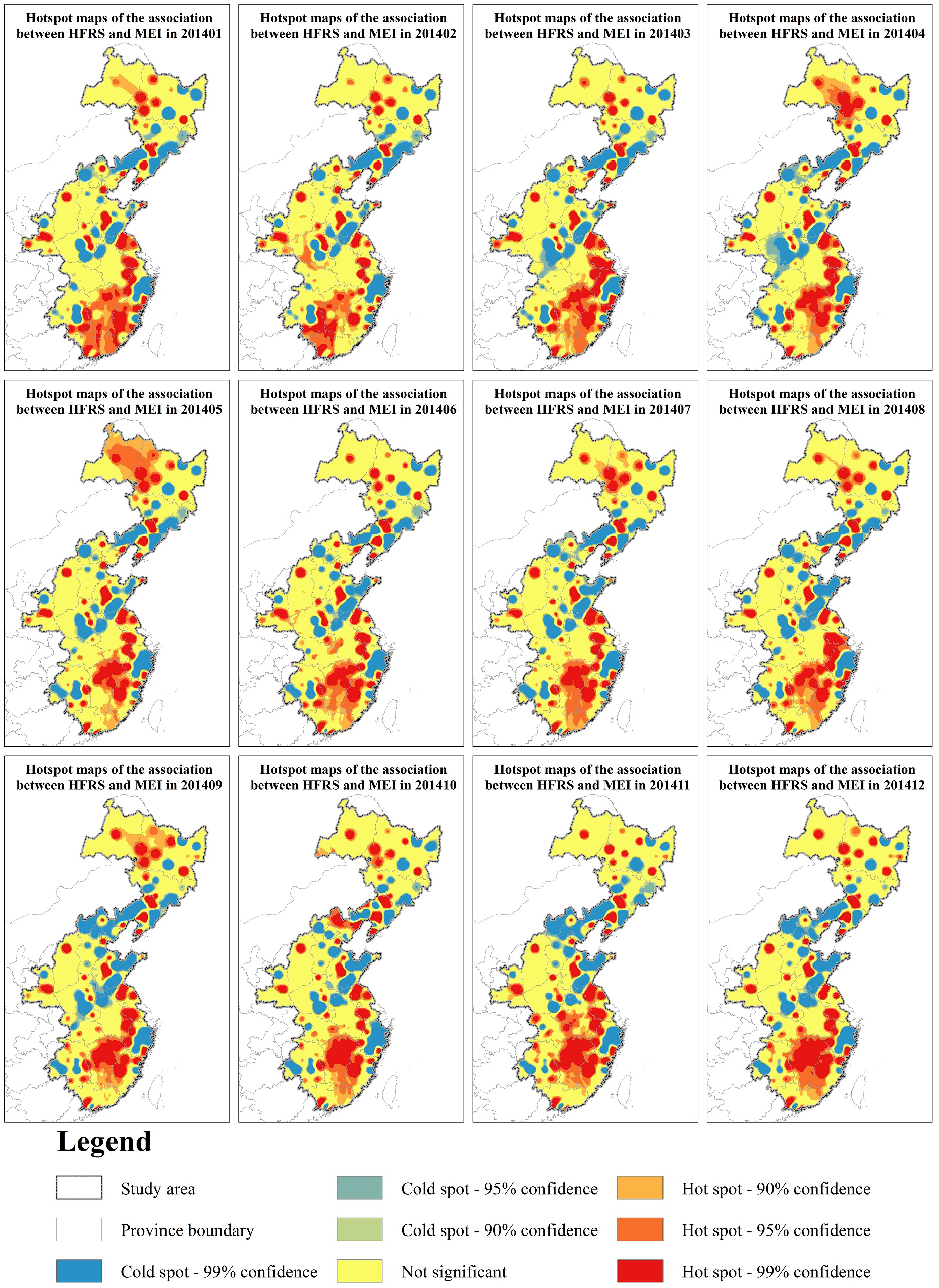

Supplement: S28 Fig — (TIF) [file pntd.0006554.s030.tif]

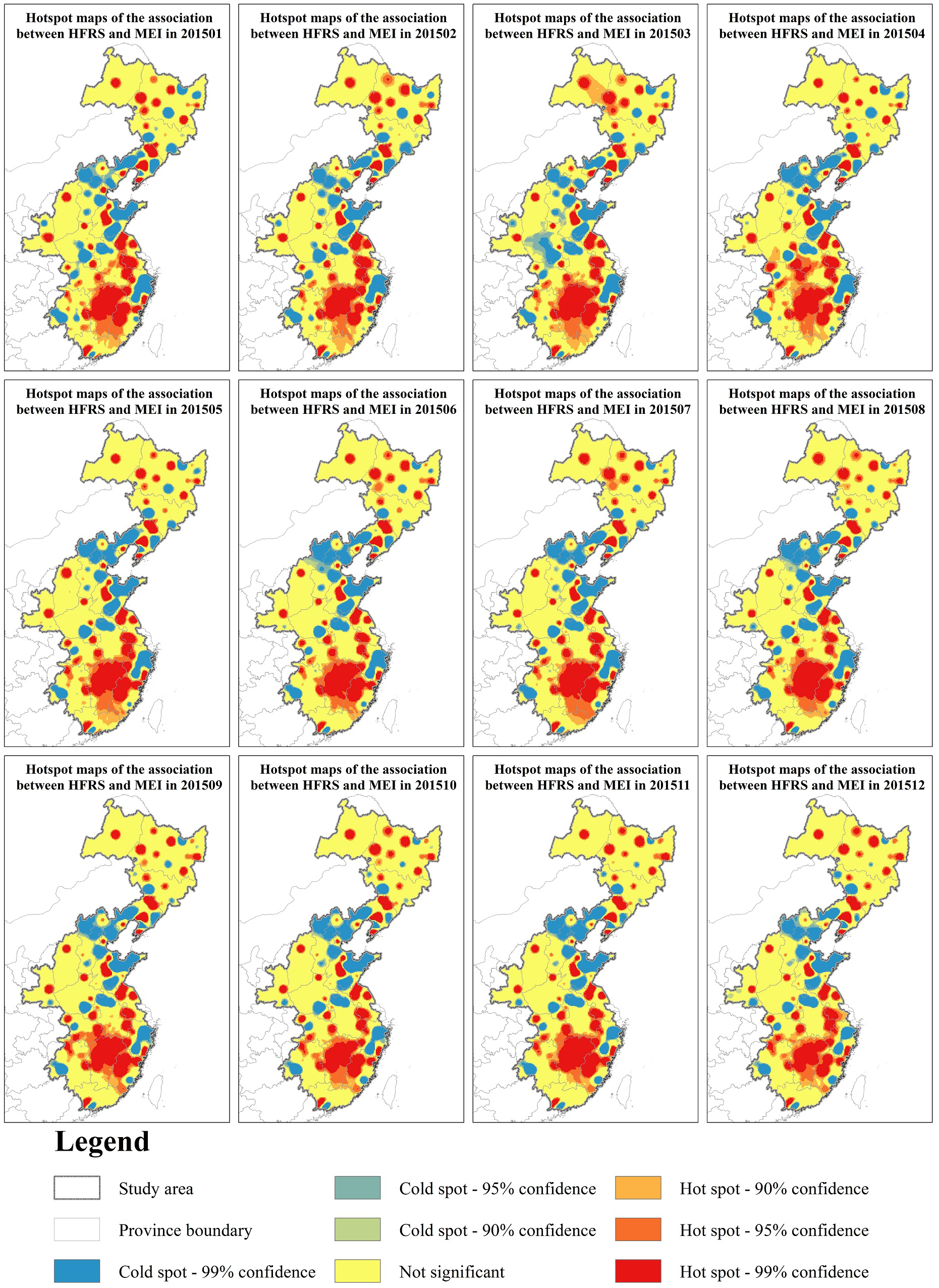

Supplement: S29 Fig — (TIF) [file pntd.0006554.s031.tif]

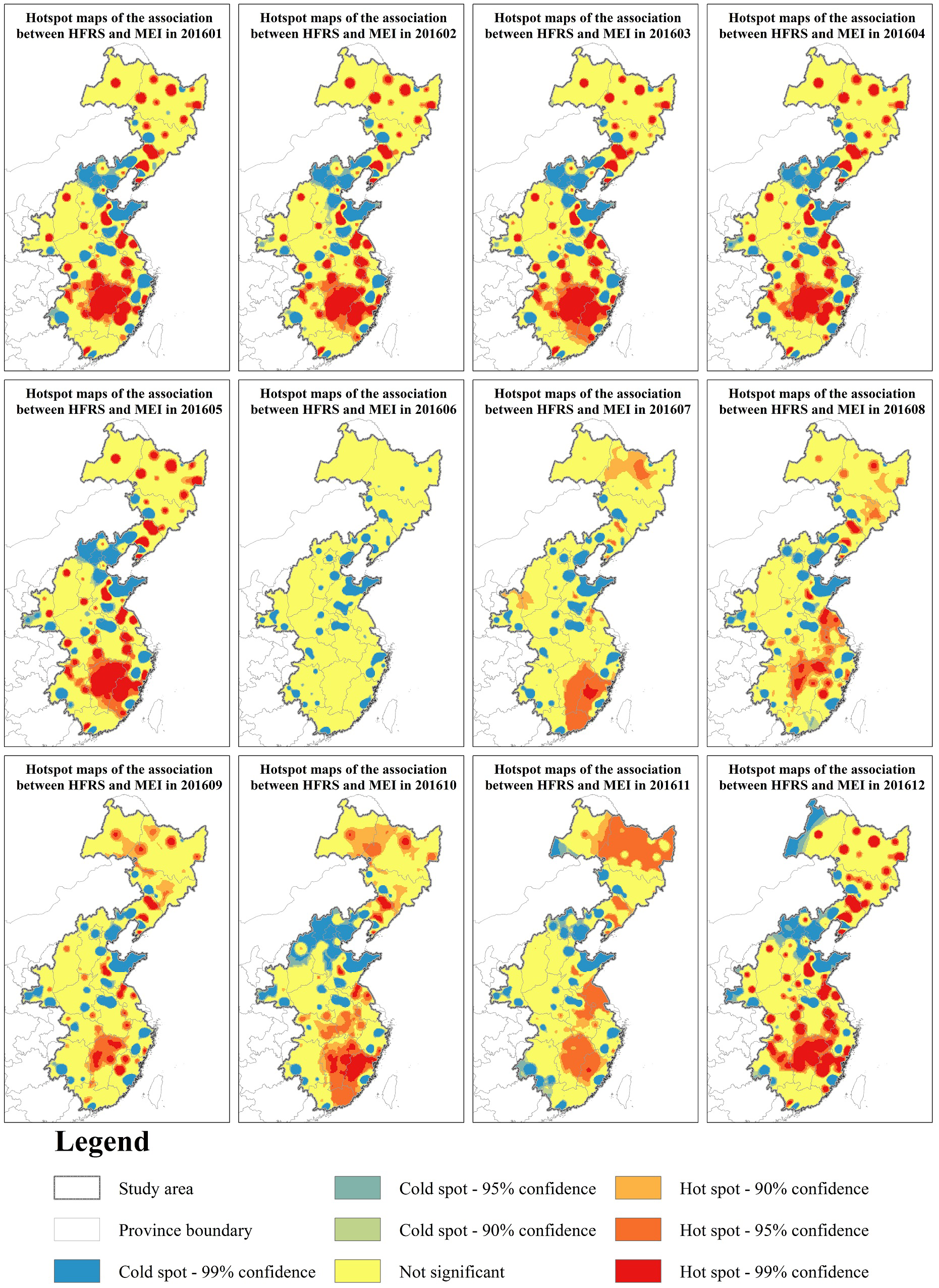

Supplement: S30 Fig — (TIF) [file pntd.0006554.s032.tif]

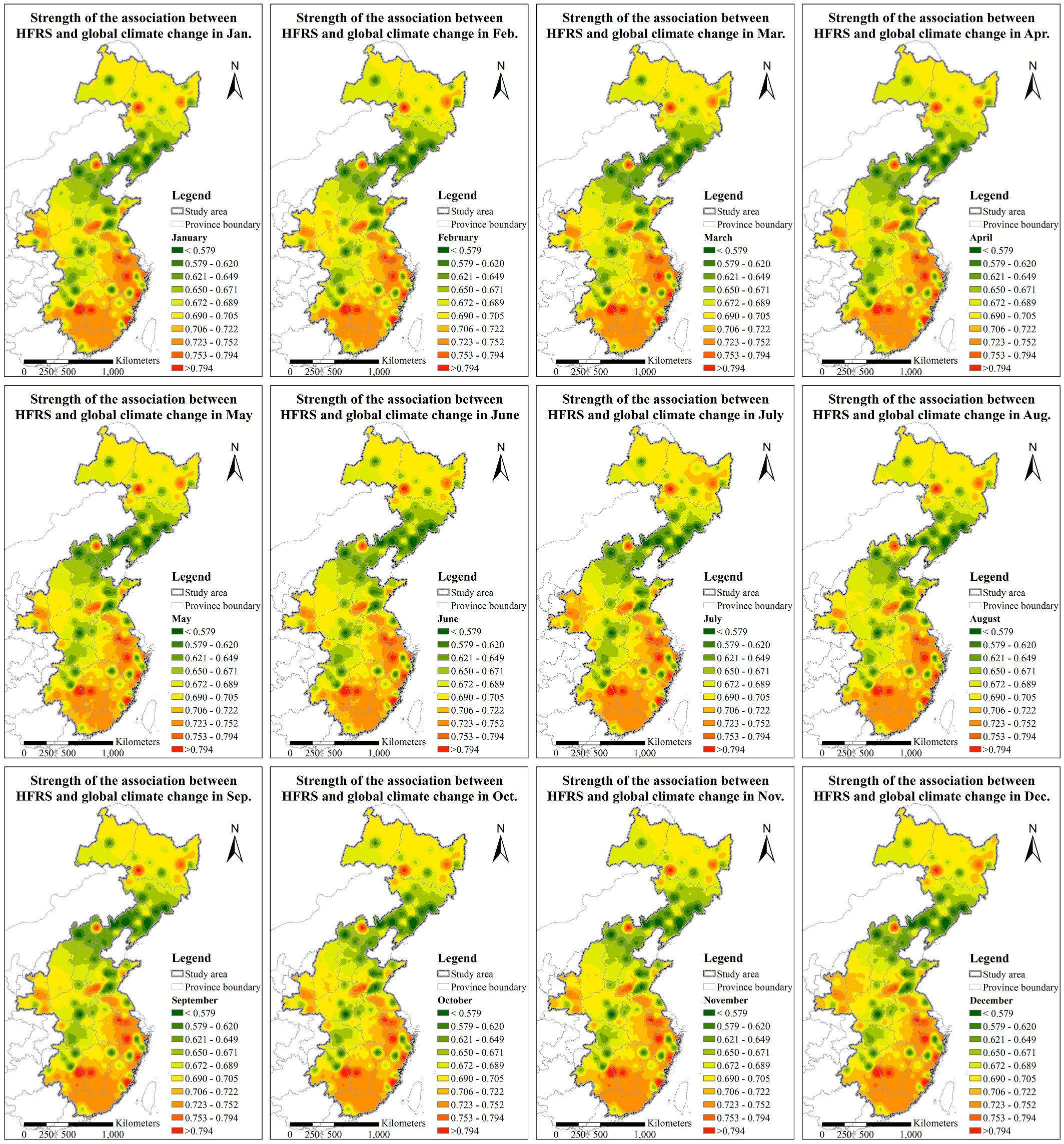

Supplement: S31 Fig — (TIF) [file pntd.0006554.s033.tif]

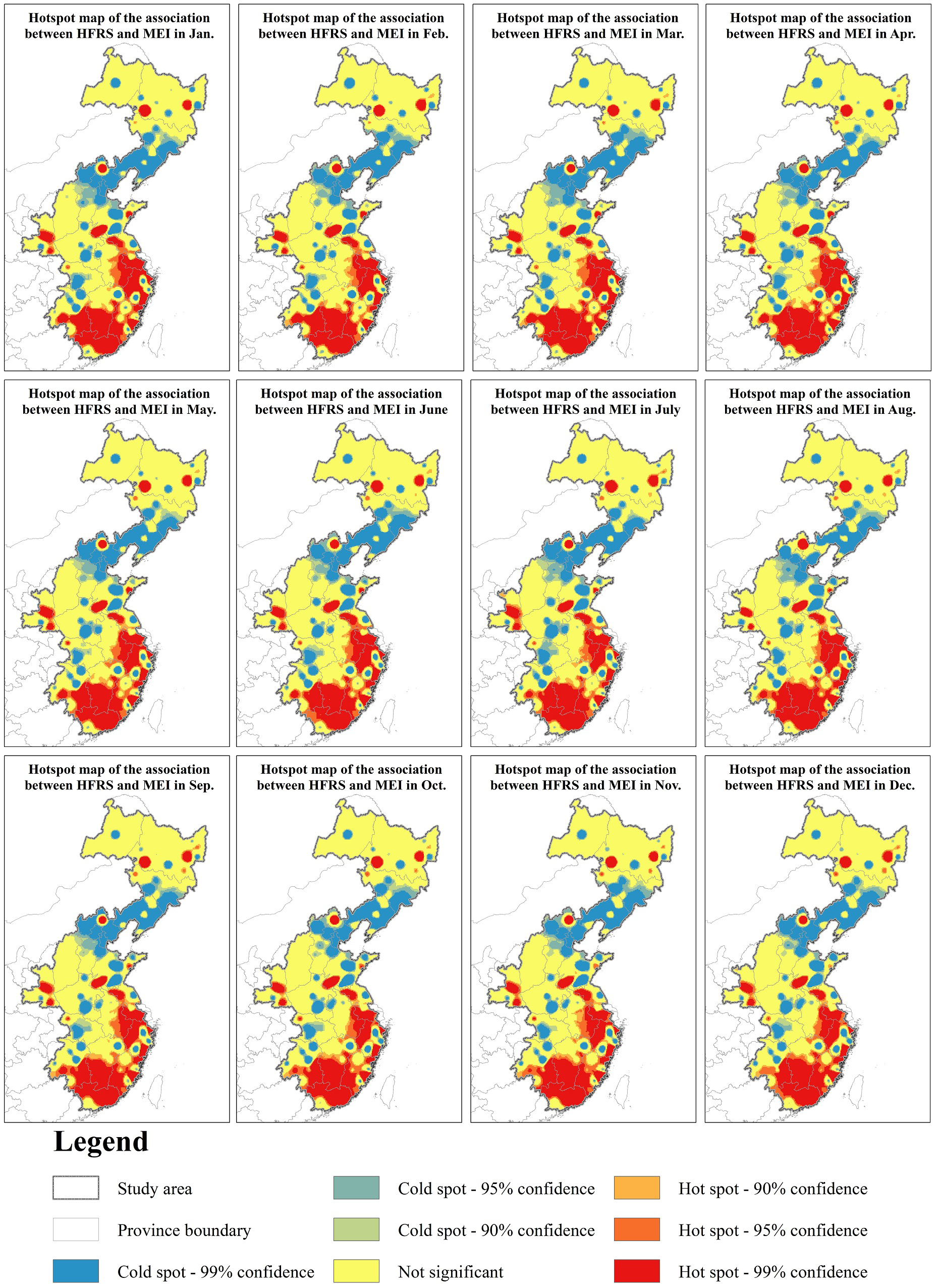

Supplement: S32 Fig — (TIF) [file pntd.0006554.s034.tif]

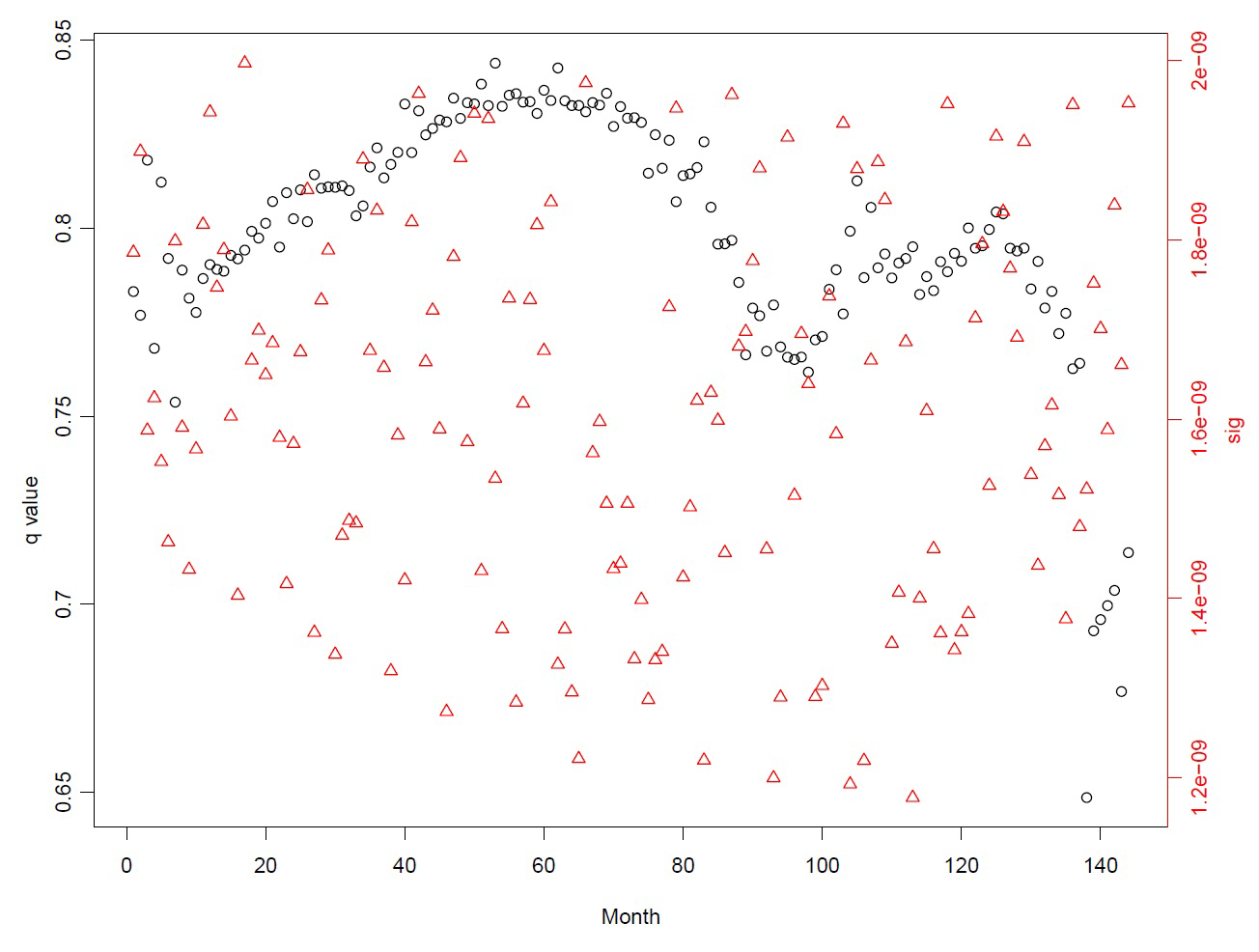

Supplement: S33 Fig — (TIF) [file pntd.0006554.s035.tif]

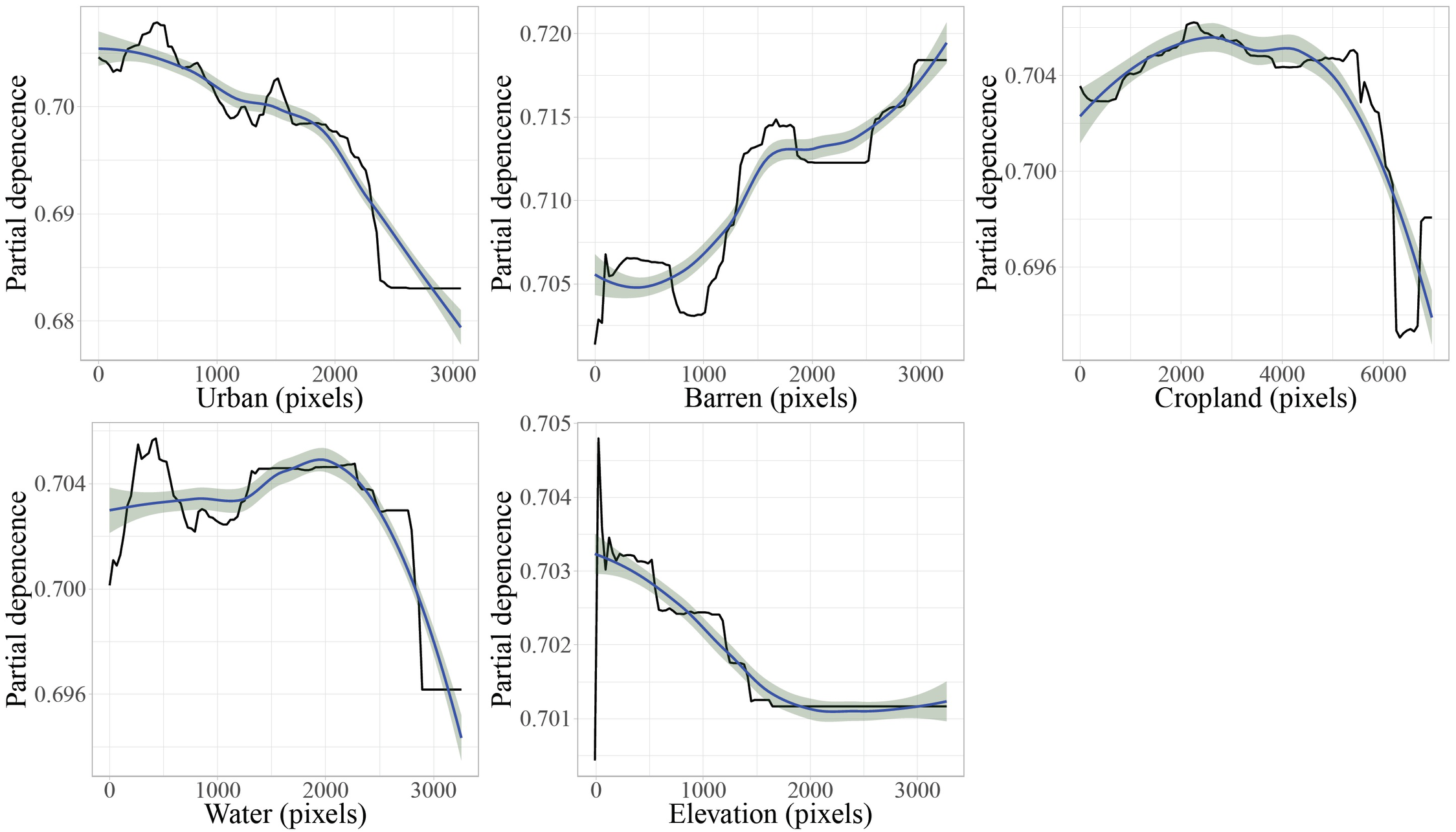

Supplement: S34 Fig — (TIF) [file pntd.0006554.s036.tif]

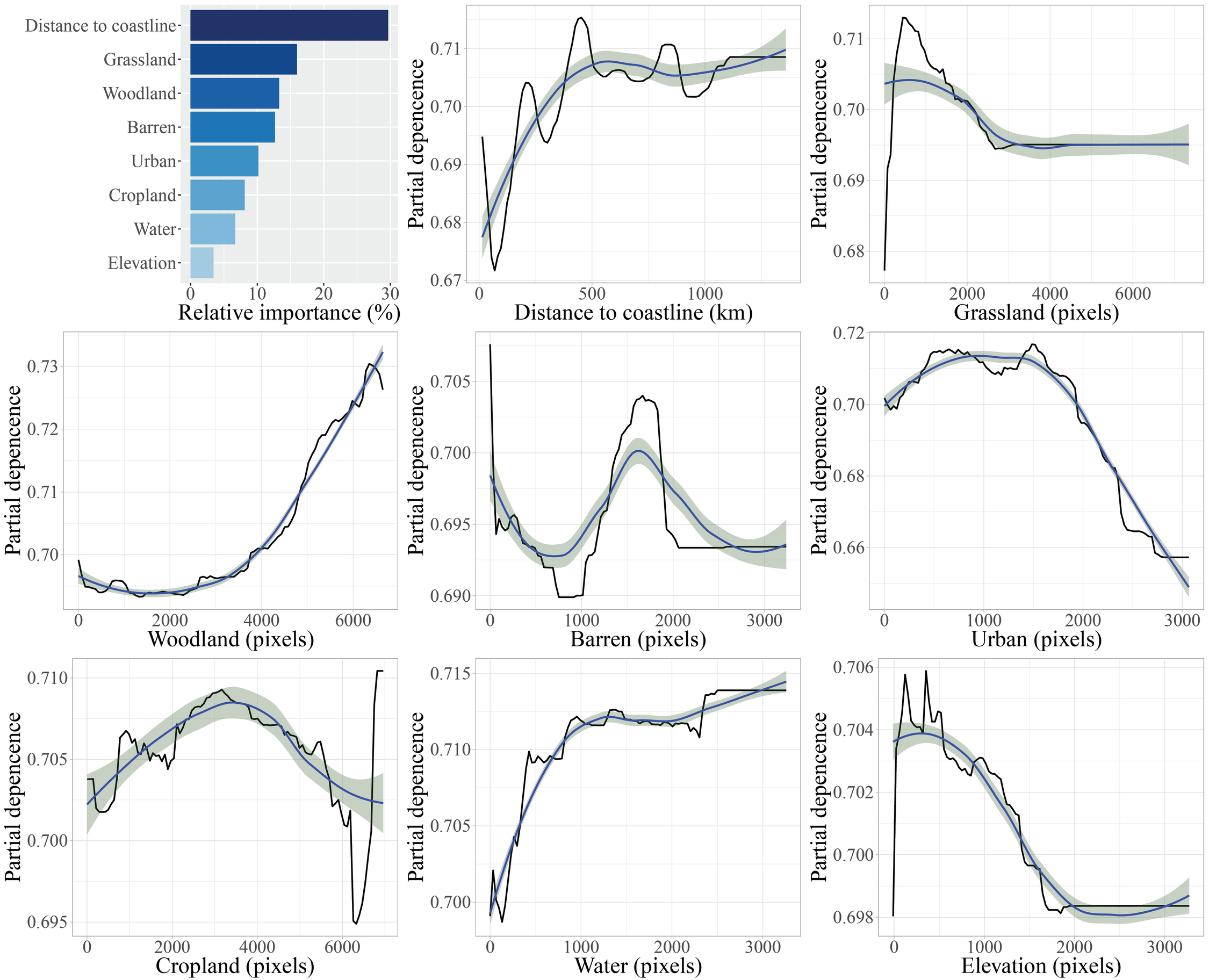

Supplement: S35 Fig — (TIF) [file pntd.0006554.s037.tif]
